# Bio‐Based Wax Interfaces for Droplet Energy Harvesting at Fluoropolymer‐Like Output Levels

**DOI:** 10.1002/advs.202515266

**Published:** 2025-11-10

**Authors:** Behnam Kamare, Mahla Shahabi, Matteo Carpi de Resmini, Tiago Fernandes, Fabian Meder

**Affiliations:** ^1^ Surface Phenomena and Integrated Systems The BioRobotics Institute Sant'Anna School of Advanced Studies Pisa 56216 Italy; ^2^ Graduate School of Life Sciences, Master’s Programme in Bio‐Inspired Innovation Utrecht University Utrecht 3584 CH The Netherlands

**Keywords:** biodegradable materials, contact electrification, energy‐autonomous devices, soft electronics, surface charging, triboelectric nanogenerators

## Abstract

Droplet impact and rebound on solid surfaces has emerged as a promising method for energy harvesting, typically demonstrated using fluorinated polymers that generate high voltages via liquid–solid contact electrification. However, these materials are non‐degradable and environmentally unsustainable. To address this limitation, bio‐based waxes ‐ selected by their potential role in environmental electrification processes ‐ are explored as sustainable alternatives. Voltage, current, and charge generation are systematically analyzed from water droplets impacting wax‐coated surfaces. Remarkably, natural waxes such as beeswax, operculum wax, and epicuticular plant waxes produced peak voltages up to 500 V and comparable current levels (≈20–40 µA, 10–20 mW peak power) to fluorinated materials under identical conditions. Building on these findings, a flexible, modular, and biodegradable droplet energy harvester is designed using zinc electrodes and wax‐coated electrification sites. By guiding droplets through predefined sliding paths and gates, multiple energy harvesting events per droplet are achieved. These results demonstrate that high‐performance droplet energy harvesting is possible using sustainable materials and tunable harvester design. Additionally, they reveal the need for further investigation of the liquid‐solid electrification mechanism on non‐fluorinated surfaces, both in engineered systems and in nature.

## Graphical abstract

Replacing unsustainable fluorinated polymers in droplet‐based energy harvesters is usually limited by low power outputs, but carefully selected bio‐based polymers are capable of creating fluoropolymer‐like voltage outputs.

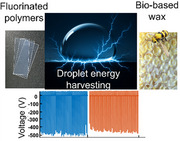

## 1 Introduction

The next generation of sustainable and energy autonomous devices, from sensor networks to robotics,^[^ ^1^, ^2^, ^3^, ^4^, ^5^ ^]^ require the capability to harvest at least part of their energy needs from their surroundings.^[^ ^6^ ^]^ Within the diversity of different energy harvesting methods, systems to create considerable electricity from single water droplets have recently impressively evolved:^[^ ^7^, ^8^ ^]^ in such devices, when a water droplet contacts a surface, electricity is generated due to contact electrification and charge transfer at the interface,^[^ ^9^, ^10^ ^]^ a phenomenon utilized in droplet‐based triboelectric energy harvesting systems.^[^ ^11^ ^]^ Droplet energy harvesters can directly drive light emitting diodes as proof‐of‐concept energy conversion,^[^ ^12^ ^]^ rain sensors, chemical sensors^[^ ^13^ ^]^ up to wave energy harvesting.^[^ ^14^, ^15^ ^]^ Usually, such energy harvesters utilize the liquid‐solid contact electrification of fluorinated polymers such as polytetrafluoroethylene (PTFE) and fluorinated ethylene propylene (FEP) as charge generation mechanism and droplet dynamics such as rebound or sliding as driving force for charge separation.^[^ ^11^, ^16^ ^]^ Fluorinated polymers were found to charge particularly high and to effectively transfer charges between the droplet and the hydrophobic surfaces. The fluorinated surface typically develops a negative surface charge while the droplet charges positively. The process generates substantial voltages, that can reach up to hundreds of volts and even kilovolts generated upon interaction of the surface with a single water droplet.^[^ ^7^, ^11^, ^17^ ^]^ The power output scales with the surface area employed for energy harvesting and the droplet falling height, volume, and number. A critical point remains: while fluorinated polymers, provide structures that resist harsh environments, these polymers are non‐biodegradable, environmentally persistent, and associated with ecological risks, which significantly constrain their sustainable and environmental applications causing the discussion of a restriction or ban of per‐ and polyfluoroalkyl substances (PFAS) based polymers.^[^ ^18^ ^]^ Soft and sustainable robotics and sensors^[^ ^19^, ^20^ ^]^ ‐ particularly those for environmental monitoring ‐ could greatly benefit from harvesting methods capable of extracting energy in natural ecosystems, e.g., from rainwater.^[^ ^21^ ^]^ However, alternative, sustainable and degradable materials^[^ ^22^ ^]^ that could replace fluorinated polymers while bearing a similar energy conversion capability are currently missing,^[^ ^23^, ^24^ ^]^ also because the liquid‐solid contact electrification and energy conversion mechanism is still under investigation and not fully understood.^[^ ^9^, ^10^ ^]^ Consequently, for water droplet‐driven energy harvesters, no effective alternative to fluorinated polymers like FEP or PTFE exist.

Here, in response to this critical gap, our research targeted how a bio‐sourced material could replace fluorinated polymers for droplet energy harvesters with very promising results. Our material choice focused on natural waxes and is based on the following assumptions. 1) natural waxes in general have an adequate hydrophobicity required to enable droplet rebound after hitting and spreading on the surface, required for effective energy conversion.^[^ ^25^, ^26^, ^27^ ^]^ 2) natural waxes have been used in solid‐solid triboelectric energy harvesters with the possibility for mechanical, for example for acoustic^[^ ^28^ ^]^ and wind energy harvesting, but not for droplet energy harvesting. Importantly, 3) we focused our material choice based on known electrical phenomena occurring in nature. We used two different types of beeswax for the reason that bees are known to be capable to detect static electric fields and use it as communication matter, even if not directly connected to a droplet‐electrification event.^[^ ^29^, ^30^ ^]^ Moreover, we used plant epicuticular waxes, as we previously showed that the epicuticular wax layer contributes substantially to currents generated in liquid‐electrification when water droplets hit plant leaves.^[^ ^16^ ^]^ Another material was pine resin, a material that was reported in its fossilized form amber as the first known material prone of significant contact electrification and triboelectric charging. Through a systematic comparison of droplet electrification through dynamic voltage, current, and charge‐transfer measurements on the fluorinated polymer FEP and on the natural waxes, we show here the first time that these waxes exhibit significant outputs upon water droplet impact reaching repeatedly and reliably several hundreds of volts, tens of µA currents, and power peaks of tens of mW, in a very similar order of magnitude of FEP, suggesting their strong potential as environmentally benign replacements with comparable performance to conventional fluoropolymer‐based systems. We then used the waxes to suggest biodegradable droplet energy harvesters that can be even further tuned by tailoring droplet sliding paths that lead to a multiplication of sequential energy conversion spots hit by the same droplet. Our results point out the opportunity for expanding the catalogue of materials in droplet energy harvesters and, moreover, suggest that electrification phenomena in nature should be further explored as they could lead to new approaches for energy conversion, harvesting and autonomous environmental sensing.

## 2 Results and Discussion

### 2.1 Material Overview and Key Characteristics

In all our experiments we used a 0.15 µm FEP film as “gold standard” for droplet electrification and comparison with bio‐derived materials in the experiments. Operculum beeswax (OBW, the specific wax that closes the hexagonal honeycombs of bee nests) and cell honeycomb beeswax (CBW), where sourced from a local farm; epicuticular plant waxes, i.e., carnauba wax extracted from the leaves of *Copernicia prunifera*, a Brazilian palm, and pine resin from coniferous trees (colophony), were purchased. As mentioned in the introduction, these materials have been specifically selected due to potential and reported roles in electrostatic charging processes. To fabricate the layers, grains or powdered materials were distributed on an electrode in quantities estimated by the desired thickness and widths and subsequently heated to melting point with a tunaable heat fan to obtain a homogenous film through recrystallization at ambient temperature (24 ± 2 °C).

**Figure 1:**
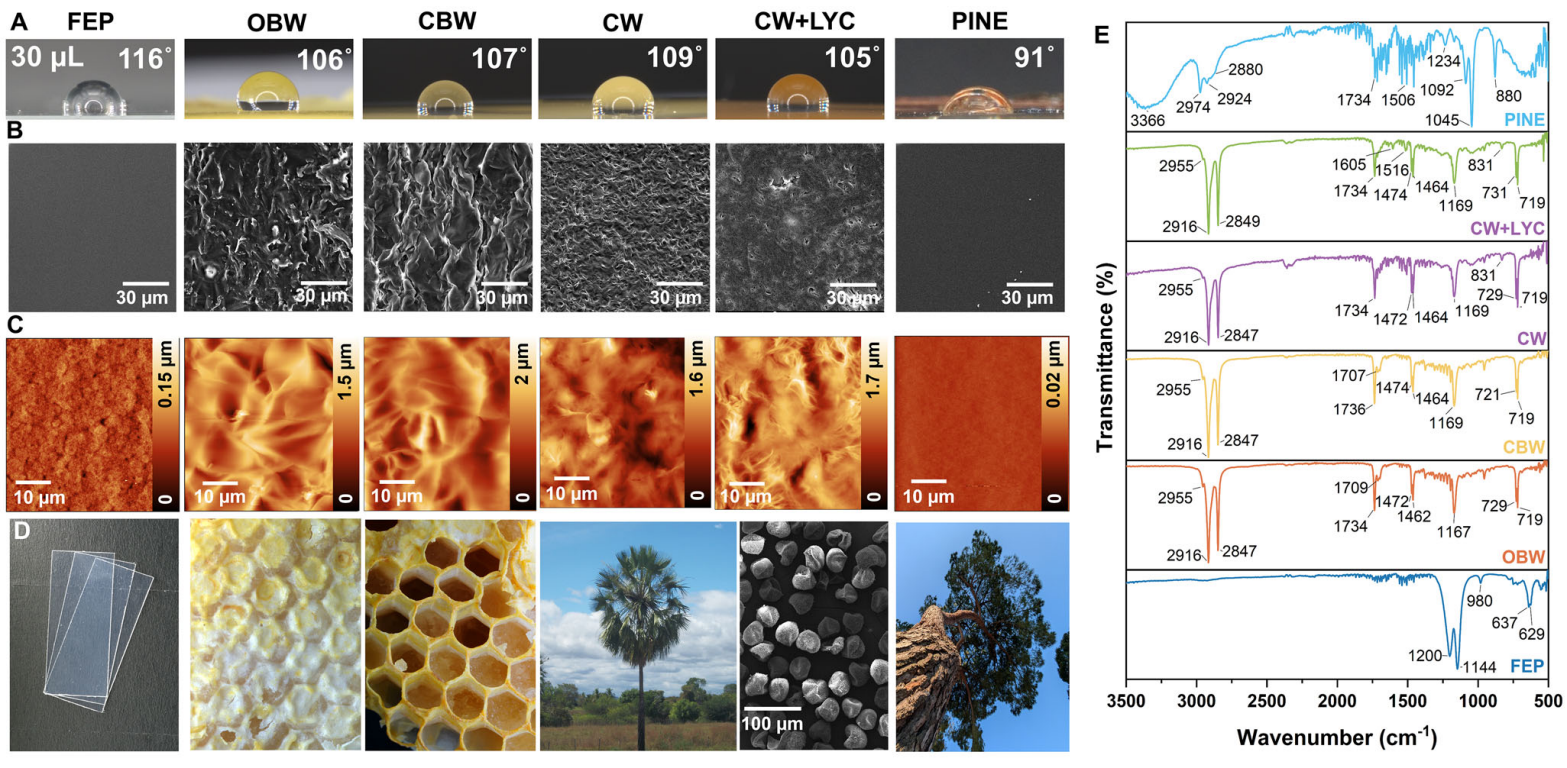
Material overview and characterization. A) Images of a 30 µL water droplet sitting on the material surface and contact angles (see measurements in Figure S1, Supporting Information) for fluorinated ethylene propylene (FEP), operculum beeswax (OBW), cell honeycomb beeswax (CBW), carnauba wax (CW), blends of carnauba wax and *Lycopodium* spores (CW+LYC), pine resin (PINE). B) SEM micrographs, scale bar 30 µm of top surface of the different materials. C) AFM topography images, scan range 50 µm. D) Photographs of the material sources, from left to right: commercial FEP sheets, bee honeycomb closed by OBW, open honeycomb showing the CBW, carnauba palm as source of CW (photo by Ouriço, 2011, image reproduced from Wikimedia Commons, public domain. https://commons.wikimedia.org/wiki/File:Carna%C3%BAbas_na_caatinga.jpg), SEM image of single *Lycopodium* spores that are part of the CW+LYC composite, and image of a coniferous tree as origin of PINE resin. E) FTIR‐ATR spectra of the materials after substrate formation.

Static contact‐angle measurements (**Figure** **1**A; Figure S1, Supporting Information) show that all test surfaces are hydrophobic, with water contact angles well above 90°. FEP, the synthetic benchmark, displays the highest angle at 116 °, reflecting its very low surface energy. Among the natural candidates, OBW (106 °) and CBW (107 °) are nearly as hydrophobic as FEP, while CW is only slightly higher (109 °). Adding superhydrophobic particles like pollen or *Lycopodium* spores during layer fabrication could be a way to further increase the contact angle,^[^ ^31^ ^]^ but we found that the surface modification was unstable during water contact and the particles washed from the wax out during water exposure. Further tuning of surface binding would be required to obtain a more hydrophobic sample. It should be noted that the pure wax layer without spores remained stable under extended tests with a high number of droplets (>11000, Figure S2, Supporting Information). When the spores are immersed in the CW (CW+LYC), this modestly lowered the angle to 105 °, indicating that the spores do not significantly alter overall wettability when they are immersed. However, it improved the ductility and crack formation in CW during cooling. Instead, pine resin records the lowest angle (91 °) but still lies in the hydrophobic regime. These values confirm that wax‐ and resin‐coated samples present water‐repellent surfaces comparable to, or only marginally less hydrophobic than, FEP expectedly assuring sufficient rebound during dynamic droplet interaction and liquid‐solid contact electrification.

To better understand the process of substrate formation with the different materials (i.e., heat treatment and recrystallization), SEM and AFM images were obtained and are detailed in Figure 1B,C, respectively. Figure 1D shows images of the material sources. Further SEM images and photographs of the samples are given in Figure S1 (Supporting Information). The pristine FEP film (control sample) displays a relatively smooth surface with a low roughness in the range of 75 nm. The OBW and CBW substrates present superimposed plates that likely formed as a function of the heat treatment and the heterogeneous/amorphous nature of its chemical composition.^[^ ^32^ ^]^ This plate‐like structures can also be seen in the AFM and lateral SEM images (Figure S1, Supporting Information) where some air pockets are also present in certain zones, especially in the CBW substrates. The epicuticular plant CW samples also display a rough and irregular surface attributed to the wax aggregation and recrystallization during substrate formation and drying process.^[^ ^33^ ^]^ The blends of carnauba wax and *Lycopodium* spores (CW+LYC) resulted in smother surface compared to pure CW. The lateral SEM images clearly show the presence of the spores deeply embedded along the carnauba wax network. PINE displayed an extremely smooth surface with at some positions some smaller crystals that expectedly formed during the drying process.

In general, the mechanical flexibility of the waxes will affect the durability and stability of the wax layer as well as the adhesion to the substrate but flexibility may also be advantageous in future applications for example in soft robotics. Previous studies report that, e.g., OBW has a relatively low Young's modulus (≈30–40 MPa) and compressive yield strength (≈0.5–0.6 MPa), with elongation at break typically in the range of 5–10%^[^ ^34^, ^35^ ^]^ but CW was significantly more brittle. As during energy harvesting, the materials are fixed on a rigid substrate, a crucial factor for energy harvesting is the adhesion of the waxes to different substrates. We applied during our tests, the waxes especially on Cu and Zn electrodes (see below) as well as on glass slides and cellulose acetate. Especially, the adhesion of the beeswaxes to those substrates was excellent as also previously observed.^[^ ^36^ ^]^ Movie S1 (Supporting Information) shows the excellent adhesion of OBW on different substrates whereas Movie S2 (Supporting Information) shows the behavior of all the tested materials on Cu electrodes, showing excellent adhesion and deformation for OBW and CBW but cracking of CW and PINE when applying bending. In any case, the adhesion and mechanical properties were sufficient to ensure stable handling and reliable electrical testing of all samples.

Next, to gain better insights in the chemical nature of the substances, FITR‐ATR data was obtained for all the different materials, and the IR spectra are detailed in Figure 1E. For FEP being a copolymer of tetrafluoroethylene (TFE) and hexafluoropropylene (HFP), where one of the fluorine atoms on TFE is replaced with a trifluoromethyl group (–CF_3_),^[^ ^37^ ^]^ the spectral bands at 1200 and 1144 cm^−1^ come from the F–C–F stretching (ν(C‐F)) vibration of TFE. The peaks at 637 and 629 cm^−1^ are attributed to the bending/deformation modes of F–C–F ((δ(C‐F)).^[^ ^38^ ^]^ The band at 980 cm^−1^ comes from the coupled stretching vibrations in the C–CF_3_ sidechain group of the HFP unit.^[^ ^39^, ^40^, ^41^ ^]^ The bio‐based polymers are of complex composition with a relatively similar FTIR spectrum across the different materials. In detail, the spectrum of OBW matches well reported data given its variable compositions of ester of fatty acids and alcohols (e.g., palmitic acid methyl ester), hydrocarbons (long chain n‐alkanes) and free fatty acids, which vary depending on the wax's origin.^[^ ^42^, ^43^, ^44^ ^]^ The bands located at 2955 (ν^as^(CH_3_)), 2916 (ν^as^(CH_2_)), and 2847 cm^−1^ (ν^s^(CH_2_)) correspond to the symmetric and asymmetric stretching of the CH_3_ and CH_2_ groups, respectively. The band located at 1734 cm^−1^ arise from the stretching of carbonyl bond (ν(C═O)) while the band at 1167 cm^−1^ is attributed to the stretching of the C–O bond from esters. Similarly, the band at 1709 cm^−1^ is assigned to the stretching of carbonyl bond from the carboxyl groups ((ν(C═O)), COOH). The 1472 and 1462 cm^−1^ band are related to the deformation CH_2_ and CH_3_, while the bands at 721 and 719 cm^−1^ correspond to the out‐of‐plane vibration of CH_2_ groups.^[^ ^44^ ^]^ As expected, CBW has a similar fingerprint IR band profile given expected similarities in chemical composition.

CW is an amorphous complex mixture of esters, free alcohols, aliphatic acids, aromatic acids, ω‐hydroxycarboxylic free acids, hydrocarbons (paraffins) and diols triterpenes.^[^ ^45^, ^46^ ^]^ Similarly to the beeswax spectra, the bands located at 2955 (ν^as^(CH_3_)), 2916 (ν^as^(CH_2_)), and 2847 cm^−1^ (ν^s^(CH_2_)) correspond to the symmetric and asymmetric stretching of CH_3_ and CH_2_ groups, respectively. The band located at 1734 cm^−1^ (ν(C═O)) is related with the stretching vibration of the carbonyl bond from esters. The weak bands in the 1600–1500 cm^−1^ region and the band at 831 cm^−1^ arise from the skeletal ring breathing modes of aromatic groups.^[^ ^47^ ^]^ The bands at 1472 and 1464 cm^−1^ are related with the deformation of CH_2_ and CH_3_ groups from the aliphatic molecules. The band located at 1169 cm^−1^ is assigned to the stretching of C─O bond from esters. The bands at 729 and 719 cm^−1^ correspond to the out‐of‐plane vibration of CH_2_ groups.^[^ ^48^ ^]^ The mixture of CW with *Lycopodium* spores (LYC) resulted in an IR spectrum mainly dominated by the fingerprint profile of the CW given its highest w/w % and the fact that the spores are deeply embedded on the wax network.

PINE is dominated by the large amount of resin acids, the broad band located in the 3366 cm^−1^ region is characteristic of the O–H stretch vibrational mode of intermolecularly hydrogen‐bonded –COOH groups. Moreover, the bands at 2974–2880 cm^−1^ are assigned to the C–H stretching vibrations of –CH_2_– and –CH_3_ groups ((ν^s^(CH_2_), ν^as^(CH_2_), and ν^as^(CH_3_)). The series of bands in the 1240–1045 cm^−1^ region arises from the O–H deformation and C–O stretching vibration of oxygen‐containing functional units and to the in‐plane C–H bending vibration of unsaturated cyclic hydrocarbons. The strong band located at 880 cm^−1^ is related to the outofplane C–H bending in aromatic rings, where its position has been previously attributed to an high content of abietic acid.^[^ ^49^ ^]^ The high complexity of the IR spectrum in the 1750–1300 cm^−1^ region, suggests the presence of oxidation byproducts of abietic acid into its most oxidized forms, such as 15‐hydroxy‐7‐oxodehydroabietic acid.^[^ ^50^ ^]^

### 2.2 Surface Electrification During Water Droplet Impact

**Figure 2:**
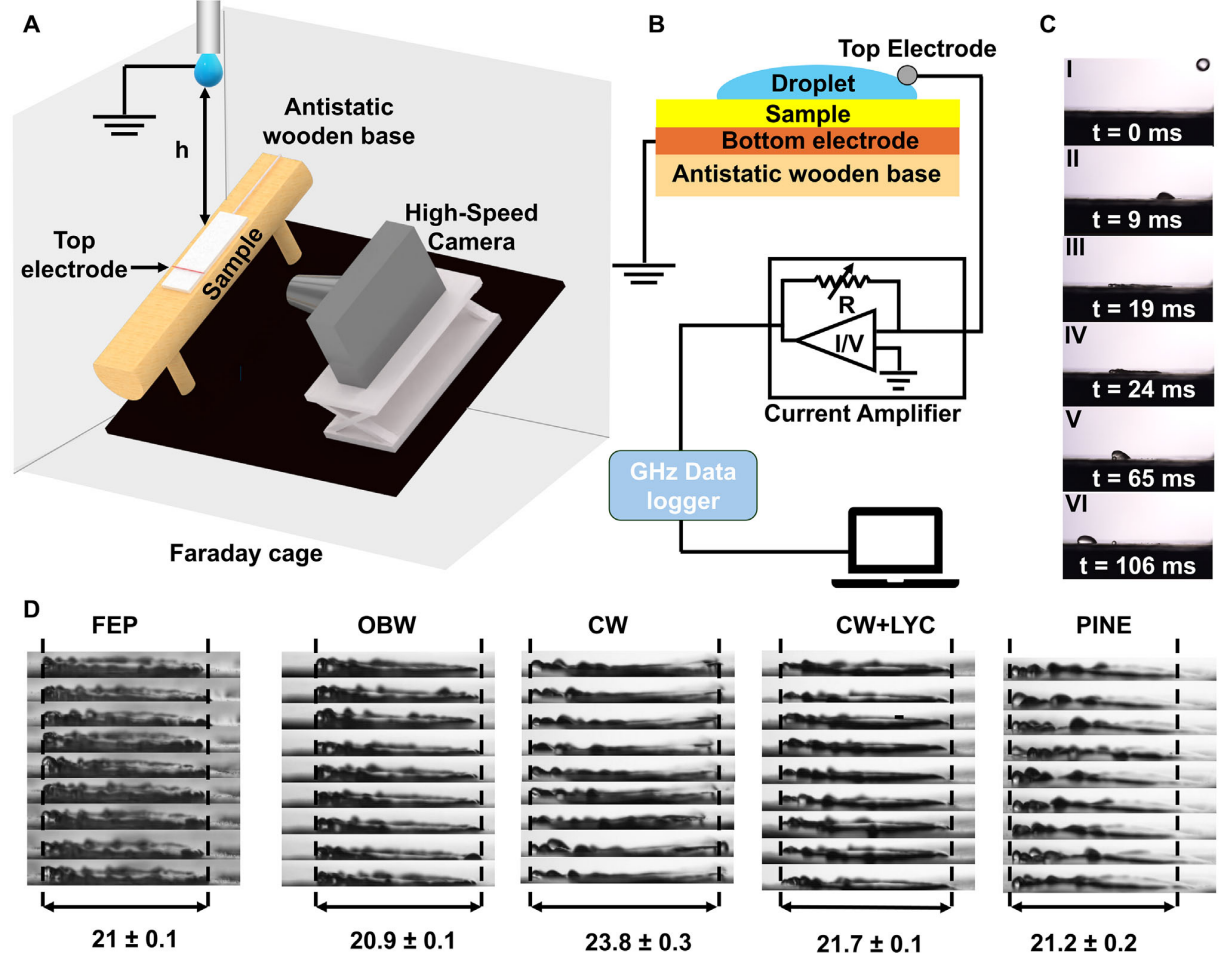
Droplet impact dynamics and setup for simultaneous electrification measurements. A) Schematic of the setup and its key components. B) Overview of the sample‐droplet multilayer forming during the measurement and the measurement circuit. C) Keyframes of high‐speed camera recordings of droplet dynamics during impact in the tilted surface (substrates are tilted at an angle of 40°, the different events highlighted as I‐VI are detailed in main text), see also Movie S3 (Supporting Information). D) Images and measurements of *n* = 9 droplets per sample at their maximum spreading width (numbers under image series are maximum widths in mm) indicating reproducible droplet dynamics.

To evaluate the water droplet‐induced electrification from falling water droplets, we conducted impact tests on the selected materials. **Figure** **2**A shows a schematic of test setup in which the sample was mounted on antistatic holder (using wood as reasonable antistatic material^[^ ^51^ ^]^). A copper electrode beneath the material layer served as bottom electrode. A second top electrode was positioned 1 mm above the sample surface. Figure S3 (Supporting Information) shows a photo of the experimental setup used for the measurements. Figure 2B gives an overview of the material structure and the setup for electrical measurements between bottom and top electrode. Water droplets with a fixed volume of 40 µL were created using a peristaltic pump and released at a frequency of ≈1 Hz onto the sample surface from a fixed height (h = 25 cm), and a high‐speed camera recorded the droplet impacts. The whole setup was mounted on tilted base (tilt angle = 40°) placed in a Faraday cage. The droplets were neutralized before falling through contacting a grounded copper shield.

Since the spreading size of the droplets upon impact is a key factor affecting contact electrification^[^ ^11^ ^]^ of the substrate and extension of the top induction electrode significantly affecting the charge transfer peak we conducted an analysis of droplet dynamics in the following. Figure 2C details the sequential phases of droplet splashing, captured by high‐speed imaging at 2408 fps. Initially, the falling droplet(I) impacts the surface (II), begins spreading (phase II), reaches maximum spreading diameter (phase III), contacting the top electrode and inducing charge transfer. From impact to the surface until reaching to the maximum spreading diameter takes ≈10 ms. Then it starts retracting (phase IV) and eventually, it forms a droplet again (phase V) that slides over the tilted surface (phase VI). Movie S3 (Supporting Information) shows the droplet dynamics.

Figure 2D instead provides snapshots from high‐speed camera videos (Movie S3, Supporting Information) at the maximum spreading phase for each tested material and 9 sequential droplets impacting the same position, quantifying the average maximum spreading diameter. All materials exhibited only very small variation in maximum spreading diameter (≈21 ± 0.2 mm), while only CW showed a marginal higher spreading diameter (23.8 ± 0.3 mm) indicating that droplet spreading width is comparable between the samples. The spreading area is ≈350 mm^2^ when assuming a circular shape. While the variation in spreading diameter and liquid surface dynamics across the tested materials was relatively small, we next examined whether the differences in the surface materials translated into measurable variations in electrical output.

**Figure 3:**
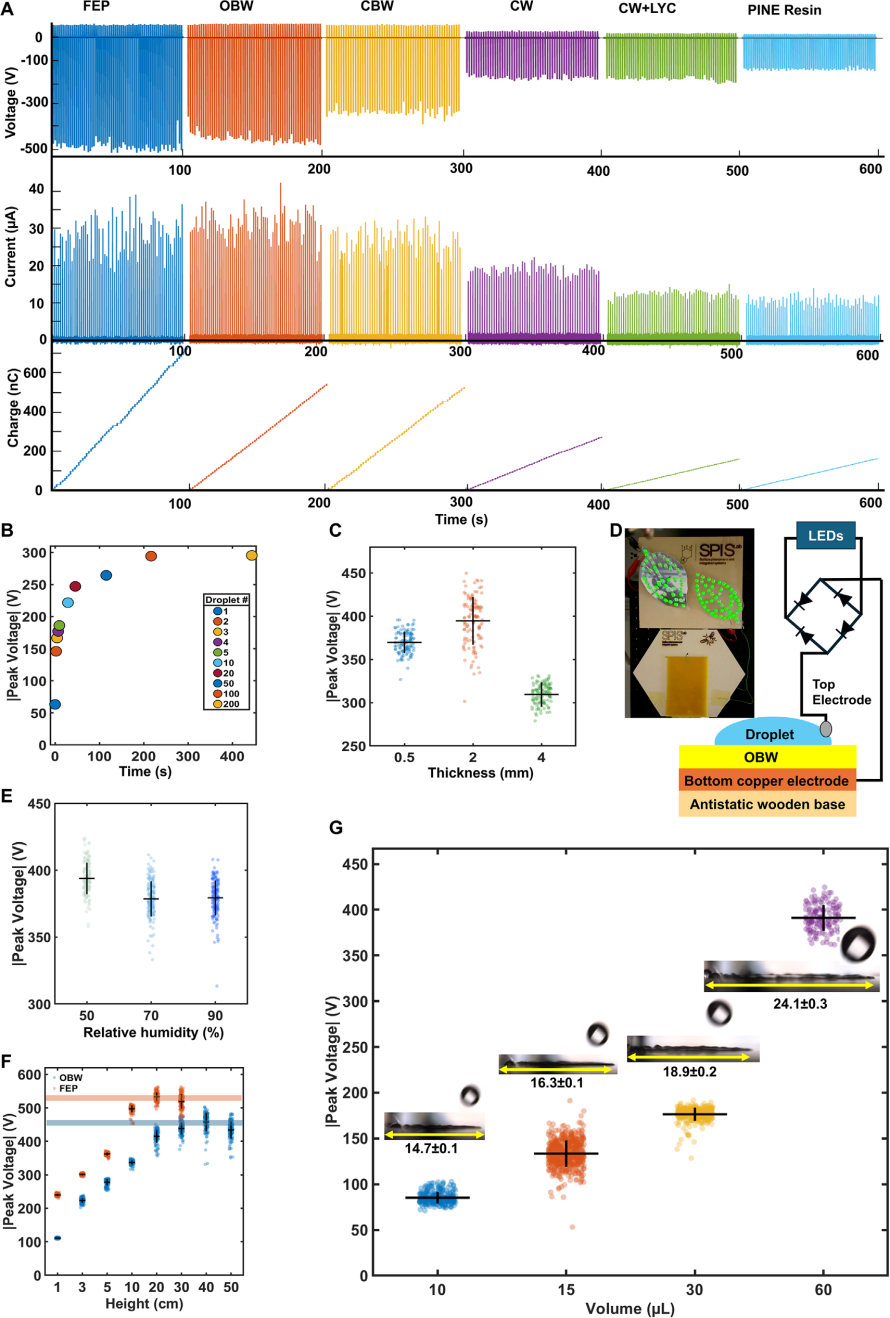
Liquid‐solid electrification of FEP and selected bio‐based interfaces. A) Saturation open‐circuit voltage and short‐circuit current peaks, and cumulative charge generated during impact of 40 µL droplets impacting the surface at a frequency of 0.7 Hz from a height of 25 cm (≈10 µJ friction‐free potential energy) using the setup schematized in Figure 2A, plots show results from ≈100 droplets hitting sequentially the samples after the surface reached charge saturation. B) Progressive charging of an OBW sample by sequential impact of 200 water droplets, saturation and maximal voltage peaks can be observed after ≈100 droplets (droplet number indicated in legend). C) Voltage and current peaks generated during impacts of *n* = 100 droplets as function of OBW layer thickness. Dots are individual measurements (jittered), thick horizontal lines are mean values and vertical bars (whisker) are equal to ±1 SD around the mean. D) Proof‐of‐concept energy harvesting prototype using OBW as electrification substrate capable to instantaneously power 100 LEDs by each droplet impact, see also Movie S5 (Supporting Information). E) Absolute peak voltage generated on OBW at different ambient relative humidities (50, 70, and 90%) and constant temperature of 23 °C. Each datapoint represents the peak voltage of an individual droplet. F) Absolute peak voltage generated on OBW as function of droplet volume (10, 15, 30, and 60 µL). Insets show frames at maximum spreading with measured spreading diameter (mean ± SD). If not otherwise stated, the experiments have been conducted at T = 23 ± 1 °C, and 50 ± 3% RH. G) Absolute peak voltage generated on OBW as function of drop height for FEP (orange) and OBW (blue) at constant droplet volume (40 µL); Lines indicate the mean voltage at highest output. *n* ≥ 100 in e), g), and f).

**Figure** **3**A shows the open‐circuit voltage, short‐circuit current, and the cumulative charge for each material under identical droplet impact conditions. Each peak in the voltage and current plots and each step in the charge measurements is caused by and indicate a single droplet landing on the surface, spreading, and contacting the top electrode. Droplet impacts on FEP caused expectedly signals of several hundred of volts (≈500 V) and current peaks in the range of 20–40 µA. Remarkably, especially OBW exhibited electrical output comparable to FEP with reproducible voltage peaks of ≈500 V and current peaks of ≈30–40 µA. The tests have been done on the same day under same conditions and at same environmental conditions (T = 23 °C, RH = 50%), samples prepared and tested at different days lead to slight variations in peak outputs still showing the same trend. This indicates that a natural wax can nearly match the performance of state‐of‐the‐art synthetic triboelectric materials. Other natural materials, such as CBW, CW, and PINE also demonstrated clear droplet‐surface electrification, although their voltage, current, and charge outputs were generally lower than those of FEP and OBW. Interestingly, the inclusion of CW‐LYC slightly decreased the current output compared to pure CW but did not significantly enhance the triboelectric performance along with the marginal effect on the contact angle.

We also investigated the sequential droplets progressively charge the OBW surface. Figure 3B shows the peak open‐circuit voltage recorded for the 1st, 2nd, 3rd, 4th, 5th, 10th, 20th, 50th, 100th, and 200th droplets exposed to the same surface at 0.7 Hz frequency. The voltage magnitude steadily increases, from 65 V at the very first impact to ≈300 V caused by the 100th drop, before levelling off between the 100th and 200th impacts. This saturation behavior indicates that each successive drop deposits additional charges onto the OBW surface until it approaches a maximum saturation charge density. This progressive charging trend is not unique to OBW and typically observed in liquid‐solid contact electrification.^[^ ^9^, ^11^, ^52^ ^]^

Another important aspect is the stability of the electrification process under extended operation and the recovery after periods of inactivity (e.g., when droplet energy harvesters should be applied outdoors without continuous rainfall). We thus recorded voltage during continuous operation for 12000 s (≈11140 droplets, ≈0.9 Hz) and we observed that it remained stable without noticeable degradation (Figure S4A, Supporting Information). After a 41 h pause, the harvester was reactivated and, following a short transient of ≈100 droplets to reach the saturation voltage, its output recovered to the same level observed before the interruption (Figure S4B, Supporting Information), demonstrating both stability and recovery in addition to the drop‐wise activation and saturation of surface charging sites. Further factors affecting energy conversion of OBW were analyzed in detail.

#### 2.2.1 Layer Thickness

Also, the sample thickness affecting dielectric strength and capacitance of the system affects the voltage output.^[^ ^53^, ^54^ ^]^ Figure 3C gives a quantitative analysis of the influence of layer thickness on the voltage peaks for OBW (current peaks and accumulative charges are given in Figure S5, Supporting Information). The sample with 2 mm thickness showed the highest average output voltage (≈400 V and more than 20 µA) while thinner sample with a thickness of 0.5 mm presented slightly lower outputs (≈375 V and slightly less than 20 µA). The thickest tested sample (4 mm) presented the lowest voltage peaks (≈300 V) while the current peaks were more than 20 µA indicating expectedly a trend toward an optimal thickness.

#### 2.2.2 Powering LEDs

To illustrate the practical potential of OBW‐based generators, we integrated the device into a simple energy‐harvesting setup including a diode bridge as shown schematically in Figure 3D. This proof‐of‐concept harvesting system, also shown in Movie S5 (Supporting Information), was capable of repetitively powering 100 LEDs each time a droplet impacts OBW confirming again that significant voltages are generated by OBW (voltage peaks of 300–500 V) and as sustainable replacements for fluorinated polymers in droplet‐harvesting micropower sources.

#### 2.2.3 Ambient Humidity

To assess the role of ambient humidity, we measured the absolute peak voltage of OBW at 23 °C while varying RH between 50%, 70%, and 90% (Figure 3E, equilibration time 10 min). Only a slight reduction (≈5–7%) in voltage peaks was observed when RH increased from 50% to 70%, but the values remained essentially unchanged between 70% and 90%. This modest decrease indicates that humidity within this range has only a minor influence on the voltage generated on OBW. We further validated this observation by performing a continuous RH sweep from 30% to 90% and then back to 30% at 23 °C. In this dynamic test, voltage peaks fluctuated within a narrow band (again less than 10% change across the full cycle), and the signal returned to its initial level once the RH was brought back to 30%. This demonstrates that the effect of humidity is reversible and that the OBW surface recovers its electrical performance after exposure to high humidity (Figure S6, Supporting Information). In contrast, varying the ambient temperature produced a clearly decreasing and still reversible effect: when the temperature was increased from 30 to 53 °C, absolute voltage peaks progressively decreased by nearly 70%, reaching a minimum during the retention period at the highest temperature. Upon cooling down to 19 °C, the signal recovered to its initial value (Figure S7, Supporting Information).

#### 2.2.4 Microstructuring

Introducing micro‐ and nanostructures is known to influence both surface hydrophobicity and triboelectric output.^[^ ^55^ ^]^ Here, we tested three different microstructrued patterns on OBW surfaces, which increased the water contact angle (Figure S8a–c, Supporting Information). However, the electrical output decreased, in some cases drastically after introducing these structures (Figure S8d, Supporting Information). This reduction is likely due to effects occurring during complex dynamics when the droplet hits the surface, partly related to a roughness‐induced droplet splashing and pinning of smaller droplets to the wax, which hinders uniform spreading,^[^ ^56^ ^]^ rebounding, and droplet shedding that are essential for efficient charge transfer. In addition, a Cassie–Baxter wetting state^[^ ^57^ ^]^ and trapped air gaps may reduce the effective solid–liquid contact area for charge transfer. Together, how tuning the structure, beyond the intrinsic micro‐nanostructure on the OBW as seen in AFM images, needs further investigation.

#### 2.2.5 Droplet Volume

To investigate the effect of droplet volume, we measured the absolute voltage peak at a fixed dropping height (20 cm) while varying the volume between 10–60 µL. The peak voltage increased with droplet volume, from ≈90–100 V for 10 µL droplets to ≈400 V for 60 µL droplets (Figure 3F). This is likely due to a combination of larger kinetic energy (10 and 60 µL droplets falling from 20 cm correspond to ≈20 and 118 µJ kinetic energy, respectively) and larger spreading area of bigger droplets, as also visualized by the spreading diameter snapshots from high‐speed videos shown above each data cluster (see also Movie S4, Supporting Information). The maximum spreading diameter, increased from ≈14.7 mm (≈170 mm^2^, assuming a circular spreading area) for 10 µL droplets to ≈24.1 mm for 60 µL droplets (≈455 mm^2^ circular spreading area).

#### 2.2.6 Droplet Falling Height

To evaluate the role of droplet falling heights, we measured the absolute peak voltage generated by 40 µL droplets impacting OBW and FEP surfaces from increasing heights (1–50 cm, 4–200 µJ, respectively Figure 3G). For OBW, absolute voltage peaks rose from ≈110 V at 1 cm to ≈430–480 V at 40 cm (orange line), reflecting a strong dependence on the falling height and related droplet kinetic energy. FEP produced consistently higher voltages even at low heights (≈230–250 V at 1 cm) and reached ≈500–530 V by 10 cm (blue line), with only little further increase at greater heights. At maximum heights, OBW becomes comparable to FEP, with peak voltages only slightly ≈10% lower; however, OBW requires significantly higher impact energy (i.e., greater falling height) to reach this level of performance. These findings suggest that OBW is more impact‐energy dependent, while FEP rapidly saturates at high output even under modest droplet heights. We also note that the standard variation of the voltage peaks increases with height. This is likely due to stronger fluctuations in droplet spreading and breakup and more complex local impact dynamics at higher kinetic energies.

#### 2.2.7 Contact Mode

To further investigate how the materials charge under different droplet contact modes, we controlled the droplet dynamics and measured the current in situ during two specific interactions: when the droplet slid across the FEP or OBW surface, and when it was repeatedly “tapped” at the same position by lifting it 5 mm and reapproaching the surface (sliding and tapping modes, respectively; Figure S9, Supporting Information). Interestingly, while for FEP current signals could be recorded under both sliding and tapping conditions, OBW showed only negligible or no currents measured in these gentle contact modes. These results suggest that the charge generation observed in natural waxes under droplet impact is likely governed by a distinct mechanism, potentially activated under dynamic, high‐energy impact conditions like that of falling droplets spreading and rebounding on the surface rather than during sustained or gentle contact.

**Figure 4:**
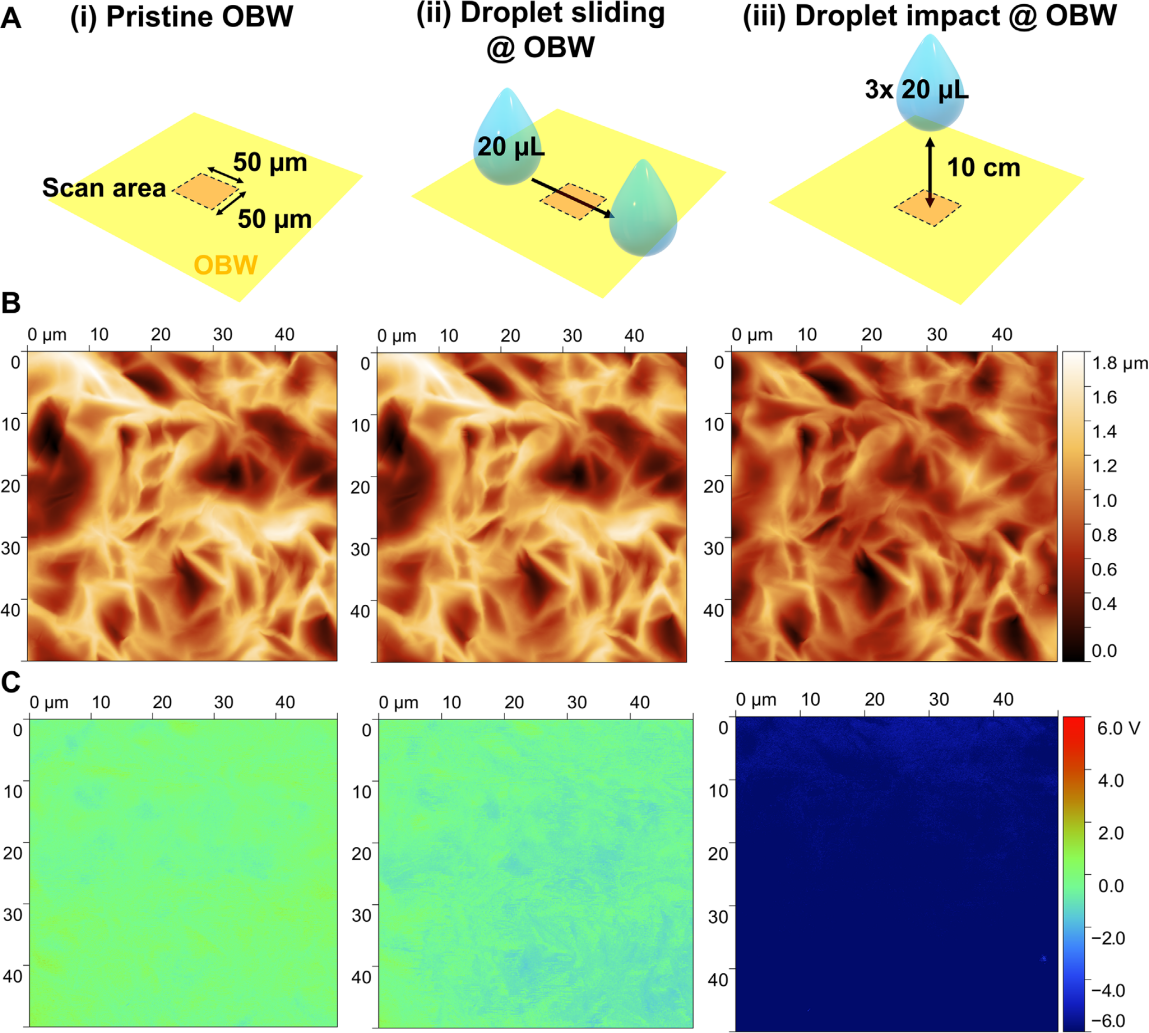
KPFM surface potential maps of OBW after different droplet–surface interactions. A) Overview of the droplet exposure protocol: (i) pristine OBW surface, no droplet exposure; (ii) the droplet was sliding over the scanning area by pulling it over the surface; (iii) the scanning area was exposed to three 20 µL droplets falling from 10 cm height. B) Topography of the samples before (left panel) and after the different droplet interactions (center and right panel) showing no significant topographical variations upon droplet exposure. C) KPFM surface potential maps: pristine surface is near neutral (left), exposure to a sliding droplet introduces negatively charges areas (middle), and after impact of falling droplets, large negative potentials dominate the field of view.

To further verify this behavior, we performed Kelvin Probe Force Microscopy (KPFM) on OBW surfaces before and after two different droplet–surface interaction which are shown schematically in **Figure** **4**A. As a baseline i), the pristine surface was measured without prior liquid contact. The second test involved a gentle contact/slide ii) where a single 20 µL water droplet was gently brought into contact with the surface and translated across the scanning area, then removed and the region was immediately rescanned. In the third test, impact iii), three 20 µL droplets were released from 10 cm above the surface and the identical region was scanned again using in each case the same scanning area and parameters (topography given in Figure 4B). The pristine OBW surface showed near neutral potentials with only small spatial variations Figure 4C. After gentle tapping and sliding, the maps displayed a slight negative shift with local contrasts within −1 to −2 V range, indicating minimal charge accumulation. However, after dropping three 20 µL droplets from 10 cm on the same location, the surface potential became predominantly strongly negative (up to −6 V). This progression again shows that OBW remains nearly neutral under gentle contact but accumulates substantial charges only under falling droplets.

### 2.3 Biodegradable Leaf‐Inspired Energy‐Harvesting Prototypes

We then aimed to translate our material findings into deployable, and fully biodegradable systems for droplet energy harvesting and designed and fabricated two bio‐inspired devices: a dual‐leaf cascade structure and a spiral droplet guide.

**Figure 5:**
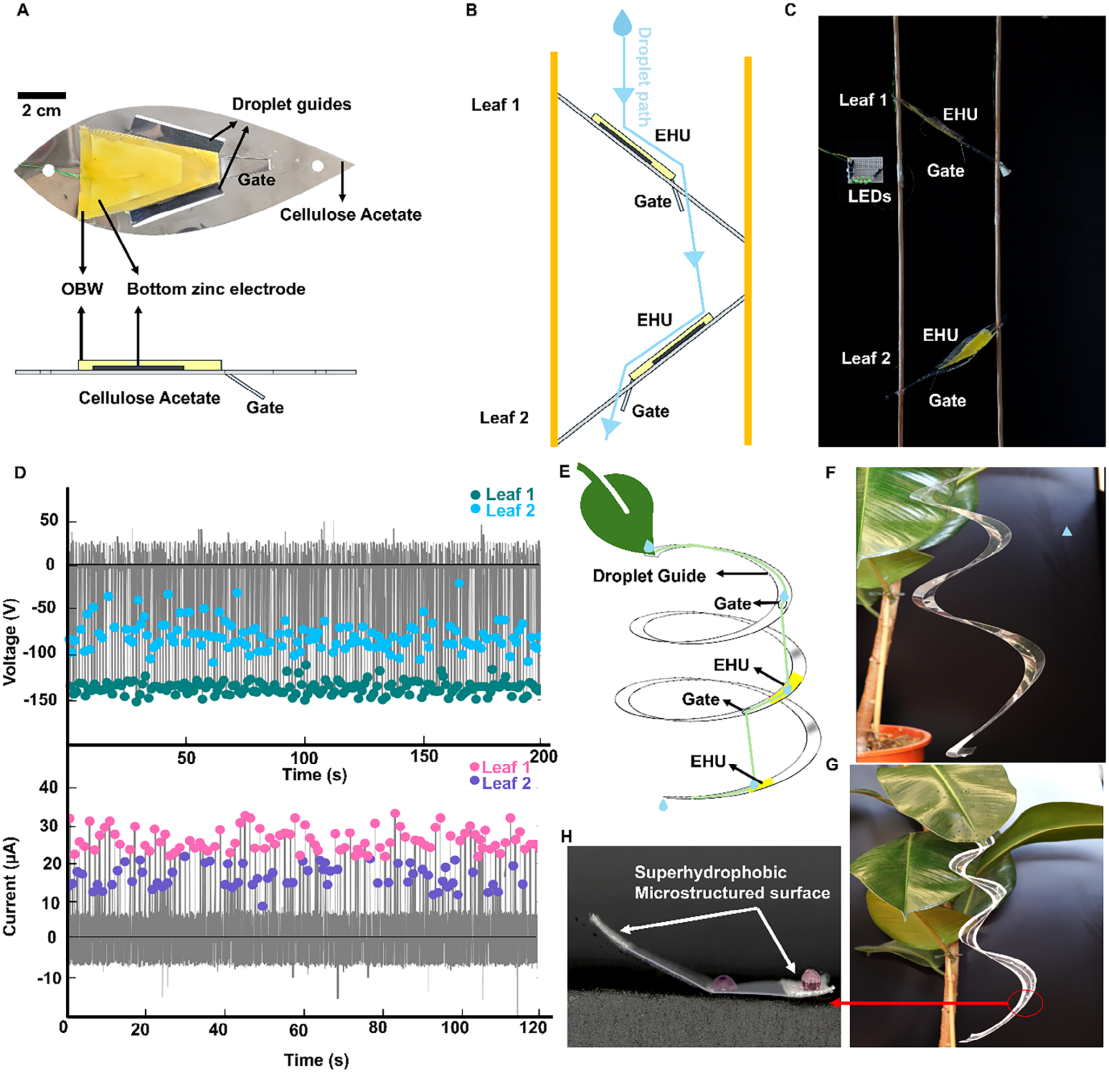
Biodegradable and multi‐modular droplet energy harvesting prototypes. A) Top‐view photograph and schematic of the side view of a leaf‐shaped droplet energy harvester of biodegradable components, OBW as electrification layer, a 80 µm zinc electrode, and cellulose acetate structural element. B) Schematic and C) photograph of the prototype of the assembly of two energy harvesting units (EHU) in a cascade manner to harvest multiple current peaks from the same droplet guiding the flow by the structure and droplet gates to the EHUs. Movie S6 (Supporting Information) shows a video of the prototype powering a 10 LED panel. D) Saturation open‐circuit voltage and short‐circuit current peaks generated by 40 µL droplets flowing through the structure and hitting the leaf 1 and 2, respectively EHUs. E–G) schematic and photographs of droplet guides made of cellulose acetate and FEP that can be clipped on leaf apex to exploit leaves as water collecting surfaces and guide droplets to an energy harvesting structure. H) in order to control droplet flow, hydrophobicity needed to be varied across the FEP structure, implementing superhydrophobic guardrails at the edges keeping the droplet in the structure.

**Figure** **5**A shows the top‐view and side view of a leaf‐shaped prototype made of OBW, a zinc electrode and cellulose acetate backbone. To guide the droplet, in a single laser‐cutting step, we laser‐cut two leaf‐shaped platforms from cellulose acetate sheets, each featuring parallel side‐cuts that funnel falling droplets into a narrow central “droplet gate.” This channelling ensures that droplets after impacting Leaf 1 are guided toward Leaf 2 for a second impact allowing for modular multiplication of current outputs from a single droplet as the droplet propagates through the prototype on multiple harvesting sites. The landing zones on both leaves were coated with OBW and backed with thin 80 µm zinc‐foil electrodes as the bottom electrode, together forming the biodegradable energy‐harvesting unit (EHU). Recently, the research on biodegradable electronics is evolving and suggesting several biodegradable electrodes for example metal films^[^ ^58^, ^59^ ^]^ like Mg, Zn, Fe, Mo, and W. We chose Zn as a proof‐of‐concept electrode material alongside OBW as its degradation products like zinc ions are crucial nutrients for plants^[^ ^60^ ^]^ when such energy harvesters are first employed and then degrade in natural ecosystems. Degradation of the electrodes and materials should be carefully tuned in detail in a future prototype for outdoor employment, e.g., to avoid toxic concentrations by controlling release and degradation rates by coatings etc.^[^ ^61^ ^]^ Cellulose acetate instead as a mechanical backbone was chosen due to a biodegradability that is potentially tunable to different environments, e.g., soil.^[^ ^62^ ^]^ As it has no direct function in the energy conversion process and only acts as structural element, it could also be replaced by other biodegradable materials. Thin zinc stripes positioned just above the OBW impact areas on both EHUs act as top electrodes, mechanically secured to detect the resulting electrical outputs upon impact. Figure 5B shows the vertical cascade arrangement in a schematic that shows the modular multiplication of energy harvesting sites per single droplet passing through the prototype. Two wooden rods support clamp Leaf 1 and Leaf 2 in a staggered, inclined stack. A droplet (light‐blue path) first impacts Leaf 1′s EHU, spreads, rebounds and subsequently leaves Leaf 1 through the droplet gate guiding it onto Leaf 2′s EHU for a second impact and energy conversion with the same droplet. Figure 5C and Movie S6 (Supporting Information) show the working prototype, in which the EHU outputs drive an LED array: each droplet instantaneously lights the LEDs multiple times. Although our demonstration uses two leaves, this approach can easily be scaled into a multi‐leaf cascade, allowing a single raindrop to produce electricity through multiple successive impacts.

Over a test using ≈200 droplets, voltage (top) and current (bottom) signals (grey) were recorded simultaneously for both leaves. Leaf 1 produced stable peak open‐circuit voltages of ≈150 V and short‐circuit currents of ≈20 µA, while Leaf 2 yielded slightly lower outputs ≈50–100 V and ≈10 µA with high variation. The colored markers represent voltage and current peaks at each sampled drop, highlighting consistent performance across many impacts. We attribute this increased scatter in Leaf 2 primarily to fluctuations in the droplet's secondary impact dynamics and landing position, which alter the effective contact area and thus the amount of charge transferred. To further validate this explanation, we performed high‐speed imaging of droplet spreading on both leaves in the prototype. High‐speed videos (Movie S7, Supporting Information) clearly show larger variability in the droplet landing position and spreading dynamics on Leaf 2 compared with Leaf 1, consistent with the increased fluctuation in the voltage output of Leaf 2. In addition, droplet charge was measured before impacting Leaf 1 and Leaf 2, respectively using a Faraday cup (Figure S10, Supporting Information). These charge measurements confirmed that droplets were nearly neutral before hitting Leaf 1 (pC range) but acquired significant charge after impacting and leaving Leaf 1 (nC range), and this initial charge on a droplet can influence the charge transfer on Leaf 2 as also observed previously.^[^ ^16^, ^63^ ^]^

Natural leaves often bear a droplet guiding structure called drip tip, which facilitate hydrophobic runoff and direct raindrops toward the leaf apex. To exploit such a feature and to develop an even lighter, rod‐free harvester that can tuck neatly under the leaf apex, we designed a V‐profile spiral channel in two material variants (Figure S11, Supporting Information). Figure 5E shows the working schematic: a natural leaf funnels droplets cross its apex into a hanging spiral with V shape profile. The spiral's flat strip is laser‐cut in one step, then folded along a shallow score line to form a V‐cross section. “Droplet gate” holes were also cut with laser cutter at specific points to allow a sliding droplet to fall through onto the next coil, creating two or more sequential impact sites per drop. We placed our EHUs directly beneath each gate so that every drop‐through produces a new contact electrification. Photographs of the two variants are given in Figure 5F,G and Movies S8 and S9 (Supporting Information). The FEP spiral (F) required an additional micro‐etched superhydrophobic “guardrail” along its edges (see close‐up in H) to prevent the high‐kinetic energy droplets from bouncing off the hydrophobic surface. By contrast, the cellulose‐acetate spiral (G) is inherently more hydrophilic, so that droplets naturally adhered and slid without any extra surface treatment while still impacting the next landing site.

In both designs, a single droplet entering at the top guide slides along the V‐track, falls through the first gate, strikes the lower coil's EHUs, and exits at the bottom. Videos of droplet sliding, bouncing and gate‐triggered drops in both spirals are provided in Movies S8 and S9 (Supporting Information). These lightweight, potentially easily clip‐on spirals demonstrate how a combination of structural design and fluorine‐free biodegradable materials like OBW can repeatedly harvest energy from single raindrops, facilitating eco‐friendly, leaf‐mounted power sources.

## 3 Discussion

We demonstrated the viability of several natural waxes as sustainable alternatives to conventional synthetic triboelectric materials especially FEP which is a benchmark in current droplet‐based energy harvesting. Remarkably, operculum beeswax (OBW), displayed electrical outputs comparable to FEP in multiple droplet‐impact tests under the same conditions. Other natural waxes and resins, CBW, CW, CW+LYC, and PINE also exhibited substantial triboelectric performance (voltage peaks >100 V), though lower than OBW and FEP. Whereas environmental conditions affected the charging behavior, the humidity variations between 35 and 90% resulted only in a small, 10 %, variation of the voltage generated on OBW and the effect was fully reversible. In contrast, temperature had stronger, still reversible effect: as temperature increases, the output dropped significantly; however, the performance can still recover after cooling down.

A potential mechanistic difference between charging of FEP and OBW is interesting and further supported by our sliding and tapping experiments (Figures S9, Supporting Information) showing clear slide‐induced charging on FEP but not on OBW under the same conditions. OBW only produced significant charge formation under falling droplets, confirmed also by KPFM surface potential maps. This suggests that OBW charging requires higher local forces from droplet impacts to activate charge transfer whereas FEP responds even to gentle mechanical excitation. Yet, even on OBW, voltages of ≈100 V when droplets fall from just 1 cm height were created and signal deviation was smallest (possibly due to more reproducible droplet spreading and breakup). Such stable behavior could be advantageous for applications requiring consistent output and compact designs.

While the FTIR spectra indicate that vibrations near ≈1170 cm^−1^ are present in OBW, CBW, CW, and FEP (the materials exhibiting the highest charging) they are less pronounced in PINE, the least charging material. However, establishing a direct correlation between these vibrations and the charging behavior remains challenging. Such vibrations are usually attributed to the stretching of the C–O bond from esters in natural polymers and to the C–F bonds in FEP but if and how these structures indeed cause the charging behavior needs further analysis. Indeed, the mechanism of charge transfer at the molecular or atomistic scale remains poorly understood and actively debated even for relatively simple polymers such as FEP, making it highly challenging to propose a molecular‐level mechanism for the chemically complex natural waxes investigated in this study. Future work should systematically investigate OBW and other wax charging at the molecular scale to identify specific structural or compositional factors that enable the highly effective liquid‐solid charge generation during dynamic droplet impacts, for example by tracking charge transfer mechanisms such as hydroxyl ion enrichment in correlation with surface chemical and structural analysis. Despite that, our results clearly confirm a) drop‐wise activation and saturation of surface charging sites, b) a droplet‐spreading area and layer thickness dependency, and c) an impact and thus contact force dependent charging that is differing from FEP.

We also explored tuning microstructure and hydrophobicity through structural modification by microstructuring (leading in our case to a reduction in voltage output due to excessive droplet pinning) and by applying *Lycopodium* spores to create superhydrophobic textures, hypothesizing increased charging when balancing surface contact and remaining surface water during rebound due to reduced wetting and rapid droplet removal (remaining water films are supposed to adversely affect the quantity of surface charge that can be induced into the backed electrode). Although initial tests successfully produced superhydrophobic surfaces, spores layers exhibited insufficient robustness, being washed away by successive droplet impacts (Figure S2, Supporting Information) emphasizing the need for more durable surface modifications to realize sustained performance gains from spore‐based modifications.

Next to material tuning, creating a reliable harvesting structure significantly affects the output performance. We build to the best of our knowledge the first droplet energy harvester with fluorinated polymer‐like output of biodegradable and resorbable materials like OBW, zinc electrodes, and cellulose‐based backbone. Metallic zinc may decompose into ionic zinc, an essential plant nutrient, cellulose acetate can be tuned to be soil‐degradable but stable in air and light, similar to OBW. A modular design allows for guiding droplets through predefined sliding paths and gates, to achieve multiple energy harvesting events per droplet, potentially multiplying the output per single droplet with each module. Yet, as we observed a decrease of the generated voltage in the second module compared to the first, further tuning, e.g., module distance and intermediate neutralization may be required to optimize outputs. Another critical challenge identified in our droplet‐impact prototyping is the inherent dependency of the electrical outputs on the droplet landing position and its local mechanical stability. Lightweight, flexible structures, such as our leaf‐inspired and spiral‐shaped biodegradable prototypes, despite their clear advantages in terms of easy integration with plant leaves, can experience inconsistent impacts, reducing electrical output stability due to environmental conditions like wind and changes in weight when some droplets might remain on the spirals. Therefore, designing prototypes that precisely guide droplets onto well‐defined and reproducible impact zones is essential, for example by guiding elements such as funnels or microstructured channels to consistently direct droplets toward best impact sites. Moreover, in cascade systems in which the droplet is recycled multiple times, proper discharging mechanisms to fully neutralize droplets before next impact may improve efficiency and stability in multiple‐harvesting scenarios.

We also want to highlight some practical considerations for a reliable fabrication of the device: The base electrode must be well insulated to prevent direct contact with droplets or humidity, which otherwise causes a significant decrease in the electrical output. In addition, the top electrode should be positioned close to the wax surface (≈1 mm) without touching it: if the distance is too large, droplets may simply slide beneath the electrode without making contact and therefore fail to discharge, while direct contact of top electrode with wax surface might also reduce the signal. The placement of the top electrode relative to the droplet landing area is also critical. If a droplet spreads on the surface too far from the electrode, or if it falls directly onto the electrode itself, the output is reduced. For stable operation, the electrode should be positioned close to but not touching the wax surface and aligned with the typical droplet landing zone to ensure consistent discharge.

Beyond showing performance comparable to that of fluorinated polymers, OBW is widely available and significantly less expensive than FEP films on a per‐mass basis (see Table S1, Supporting Information for a cost comparison from common suppliers).

Moreover, our study highlights another important finding: water–contact electrification may also occur in natural environments, generating significant voltages and electric fields. For instance, raindrops impacting leaves or the operculum of honeycombs may induce voltages with potential effects on organisms and influence the electrostatic sensing of insects. These observations underscore the need for further investigation into liquid–solid electrification on bio‐based materials, both to better understand the underlying mechanisms and to develop sustainable, nature‐inspired strategies for next‐generation energy‐autonomous technologies, sensor networks, and robotics, especially when applied in natural ecosystems.

## 4 Conclusion

In summary, this work highlights operculum beeswax as a sustainable, high‐performance, biodegradable alternative to synthetic fluorinated polymers for droplet‐impact triboelectric energy harvesting. Our results confirm a) drop‐wise activation and saturation of surface charging sites, b) a droplet‐spreading area and layer thickness dependency, and c) an impact and thus contact force dependent charging that is differing from FEP. We have shown that careful control of impact and material properties, coupled with innovative multi‐impact harvester designs, using either a cascading leaf arrangement or a spiral channel, allows a single raindrop to generate multiple times hundreds of volts. Our fully biodegradable prototypes successfully powered LEDs in real time under dripping conditions. By combining environmentally benign materials (cellulose acetate, beeswax, zinc) with straightforward precision laser‐cut guidance structures, we show that one can obtain plant‐mountable prototypes that exploit the drip off of the leaf apex for water collection to then generate electricity for low‐power devices that harness rainfall, e.g. for environmental and microclimate monitoring. Future efforts will focus on understanding the impact‐driven charging mechanism in OBW and similar materials, scaling cascade designs for higher power densities, improving design and durability while tuning biodegradability to specific application scenarios, e.g., in real‐world outdoor environments from rain droplets.

## 5 Experimental Section

### Sample Preparation

Fluorinated ethylene propylene (FEP) film exhibiting 95% optical transparency and high chemical stability were obtained from Unitak3D (model: UNI086L‐5) and utilized directly without any additional processing. Zinc films (ZincTape®, 80 µm thickness) were obtained from Metalnastri S.r.l, Italy. Operculum and honeycomb bee wax were collected from “Azienda Agricola La Valle,” a farm in the province of Pisa (43°32′5.06″N 10°43′59.18″E). The colonies were composed of typical Italian honeybees (*Apis mellifera ligustica* Spinola, 1806). The wax was naturally deposited on the wooden brood‐frames. Once ready for harvesting, they were taken out. The raw wax was separated from the operculum wax through the use of a specialized uncapping fork. The collected wax was then melted to 65 °C, facilitating separation density‐driven (impurities being heavier than both types of bee wax). Finally, residual particulate matter was mechanically scraped off.

The carnauba wax, sourced from Alteya Organics (Bulgaria), was certified organic by CERES GmbH and employed as received. *Lycopodium clavatum* spore powder was acquired from Laboratorio d'Erbe Sauro (Italy), while ecological‐grade pine resin, originating from the European Union, was supplied by Aleco. All reagents and materials were used in their as‐received state without further purification or modification.

Beeswax, carnauba wax (wax‐based), and pine (resin‐based) samples were prepared by forming a custom mold using standard microscope glass slides and copper film (35 µm thickness) that served as a mold. The samples were subsequently thermally processed using controlled heating using a tunable hot air gun (Toolcraft ZD‐8908) to obtain full melting. The specimens were cooled at room temperature and used without any modifications for further characterization and performance evaluation. To engineer hydrophobicity, *Lycopodium clavatum* spores were distributed on the molten carnauba wax as both materials were intrinsically hydrophobic or homogeneously dispersed into the molten wax matrix (an amount of spores was added so that it homogenously dispersion reached saturation, further addition lead to phase separation) before it cooled down, forming a hybrid structure with both hydrophobic and triboelectric functions.

### Electron Microscopy

Samples were mounted on aluminum stubs with double‐coated conductive carbon tape for grounding and then sputter‐coated with a thin layer of Au‐Pd to aid surface conduction and to prevent charging during electron beam exposure. SEM samples were examined using a scanning electron microscope (Quanta 400 FEG SEM, Thermo Fisher Scientific, Netherlands).

### Faraday Cage

The measurement setup was enclosed in a custom Faraday cage constructed from an aluminum frame covered with conductive mesh specific EMI shielding mesh (>60 db between 10 kHz and 22 GHz), providing continuous electrical contact across all sides (Figure S3, Supporting Information). Coaxial cables were used and routed through grounded BNC feedthroughs and connected to a data logger placed outside the cage with the same ground reference. The droplet delivery tube passed through a small hole in the conductive mesh. To ensure that the droplets were uncharged when leaving the tube, a grounded copper tube through which the droplets ultimately passed before free fall was fixed directly at the tube outlet. Control tests with short electrodes confirmed that the baseline noise inside the cage was unaffected by these entrances.

### ATR Analysis

The chemical structure of the different samples was examined by Attenuated Total Reflectance Fourier Transform Infrared Spectroscopy (ATR‐FTIR, BRUKER EQUINOX 55 spectrometer from Germany). The crystal was touched directly with each sample under constant pressure to achieve the best spectral properties. A higher signal‐to‐noise ratio and a good resolution of the characteristic vibrational bands were achieved by recording spectra in the mid‐infrared spectral region from 3500 to 500 cm^−1^, for 512 scans with a spectral resolution of 4 cm^−1^. All measurements were conducted at room temperature.

### Contact Angle Measurements

The contact angle measurements were performed using an Attension Theta Flex optical tensiometer (Biolin Scientific) to evaluate wettability on surfaces of FEP, BW, CBW, CW, CW+LYC, and PINE. The instrument was then used to dispense a 5 µL droplet of deionized water on each surface, using its dosing system. Measurements of contact angles were performed via high‐resolution side‐view pictures taken immediately following droplet deposition using the sessile drop technique and shape analysis based on the Young–Laplace fitting algorithm in the OneAttension software. The measurements were performed in a controlled air environment (22 ± 1 °C, relative humidity of 45 to 55%). The mean static contact angles of each material were determined to provide accurate characterization of surface wettability.

### Atomic Force Microscopy

The AFM measurements of all substrates were accomplished using FlexAFM™ with C3000i controller from Nanosurf operating in dynamic mode with active vibration isolation and acoustic isolation. A monolithic silicon cantilever aluminum reflective coating and 48 N m^−1^ nominal force constant (Dyn190Al) was used to collect the topography data in tapping mode. KPFM measurements were done using electrically conductive, chromium coated tips (Multi75E‐G‐10, BudgetSensors, Bulgaria). Measurements of surface potentials before and after exposure to droplets were done as follows. First, the pristine surface was measured, then the AFM head was removed from the sample and a 20 µL droplet was added at a position above the scan area and then pulled with the pipette over the scan area. Then the head was placed back in a manner that the same position as before droplet exposure could be scanned. The same was repeated after dropping 3 x 20 µL from a height of 10 cm onto the sample.

### Laser Cutting

All the patterns and designs were cut using a laser cutter (Flux Beambox Pro).

### High‐Speed Video Recordings and Imaging

All high‐speed video recordings were accomplished using the Freefly Wave continuous high‐speed video camera, operating at 2400 fps and using the objective lens from Laowa Venus Optics 100 mm t/2.9 2x Macro APO Sony E Cine. All the post processing of the images were carried out using Wave player.

### Electrical Measurements During Droplet Impacts

Current and charge signals were measured using a Keithley 6517B electrometer at 20–200 µA range connected to the PicoScope 5244D from Pico Technology. Voltages were directly recorded using the PicoScope and a 100x probe at 200 kS s^−1^. For controlled release of the water droplets, the Gilson MINIPULS 3 Peristaltic Pump was used at a operating flow of typically 20 mL min^−1^. Silicone tubing was used and attached to a metal outlet that was grounded to remove potential charges formed during liquid propagation in the tubing. All data was analyzed using MATLAB R2024b.

### Charge Measurements on Prototype

The charge state of individual droplets was measured using custom‐made Faraday cups connected to the electrometer (Keithley 6517B). The Faraday cups were placed before Leaf 1 (after the droplet separates from grounding wire in free fall) and again after leaving Leaf 1 before hitting Leaf 2 (schematic in Figure S10, Supporting Information). As droplets passed through the cup, the induced current signal was recorded using PicoScope (5244D) and integrated in MATLAB (2024b) to obtain charge values. Prior to Leaf 1, droplets were grounded and carried negligible net charges.

### Relative Humidity Control

The effect of relative humidity (RH) on the absolute peak voltage of OBW was evaluated using a benchtop climatic chamber (ESPEC SH‐242, ESPEC Corp., Japan). The chamber allows precise control of both temperature and humidity in a sealed environment. Experiments were performed at 23 °C, with RH adjusted stepwise to 50%, 70%, and 90%. At each setpoint, the system was allowed to equilibrate for 10 min before recording.

In addition to the stepwise protocol, a continuous RH ramp (30 → 90 → 30% at 23 °C) was carried out in the same chamber to evaluate the effect of dynamic humidity variation. The full trace of this experiment is provided in the Figure S6, (Supporting Information).

### Temperature Control

Temperature‐dependent measurements were performed in the same climatic chamber (ESPEC SH‐242). The chamber was programmed to increase the temperature from 30 to 53 °C, hold at 53 °C for 2 min, and then decrease back to 19 °C. Voltage responses were continuously recorded during heating, retention, and cooling phases, and the absolute peak voltages were extracted for analysis (Figure S7, Supporting Information).

### Volume Effect Test

Droplets with four different volumes (10, 15, 30, and 60 µL) were produced using nozzles formed by rolling copper tape of defined diameters. For the smaller droplets (10 and 15 µL), the narrower rolled copper nozzles were inserted inside the silicone tube, whereas for the larger droplets (30 and 60 µL), wider rolled copper nozzles were mounted externally at the tube outlet. All nozzles were grounded. At a constant volumetric flow rate supplied by the syringe pump, larger outlet diameters generated bigger droplets at a lower release frequency, while smaller diameters produced smaller droplets at higher frequency.

### Statistical Analysis

The numerical data from the electrical measurements were presented either as raw data (voltage/current/charge vs time) without further preprocessing or after extraction of the peak values through signal processing in MATLAB. Data points were typically shown as mean ± standard deviation (SD) or as point clouds, with horizontal bars indicating the mean and vertical bars representing the SD. The sample sizes (n) for each experiment were provided in the corresponding figure captions.

## Conflict of Interest

The authors declare no conflict of interest.

## Supporting information



Supporting Information

Supplemental Movie 1

Supplemental Movie 2

Supplemental Movie 3

Supplemental Movie 4

Supplemental Movie 5

Supplemental Movie 6

Supplemental Movie 7

Supplemental Movie 8

Supplemental Movie 9

## Data Availability

The data that support the findings of this study are openly available in Zenodo at https://doi.org/10.5281/zenodo.16781231, reference number 16781231.
