# Supplementary material for: Bio‐Based Wax Interfaces for Droplet Energy Harvesting at Fluoropolymer‐Like Output Levels
Source: Adv Sci (Weinh). 2025 Nov 10;13(15):e15266. doi: 10.1002/advs.202515266 (PMC13042751; doi:10.1002/advs.202515266)
Supplement: Supplementary file 1 — Supporting Information [file ADVS-13-e15266-s003.docx]

Supporting Information

Bio-based wax interfaces for droplet energy harvesting at fluoropolymer-like output levels

Behnam Kamare, Mahla Shahabi, Matteo Carpi de Resmini, Tiago Fernandes and Fabian Meder*


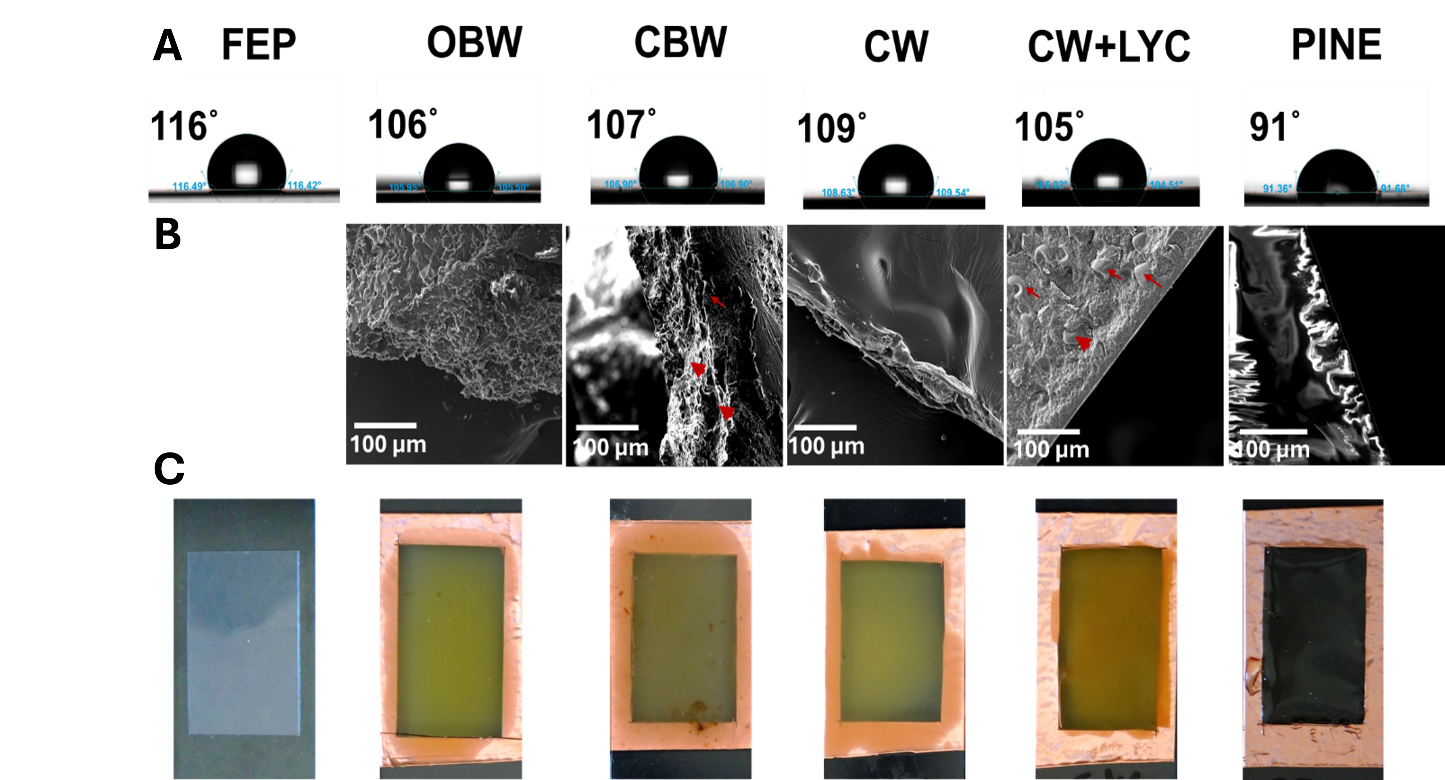


**Figure S1. Surface characterization and sample preparation of six materials.** Six different materials—FEP, OBW, CBW, CW, CW+LYC, and Pine resin—were evaluated for their wettability and surface hydrophobicity using optical tensiometry. (A) Contact angles measured from 5 µL water droplets on each surface illustrate their varying degrees of hydrophobicity. (B) SEM micrographs show both top and side views of the sample surfaces, highlighting their distinct morphological features (red arrows indicate characteristic surface textures). (C) Optical images of the fabricated samples. Thin molds were prepared by framing standard microscope slides, and the materials were thermally melted at their respective melting points to coat the mold area uniformly without altering their main structure.


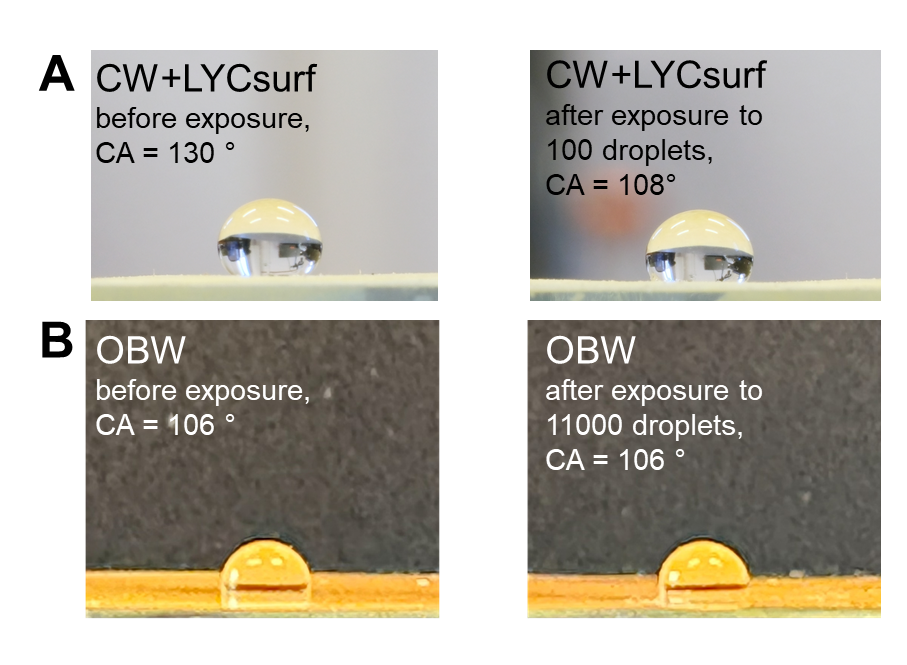


**Figure S2. Comparison of wax surface stability with and without *Lycopodium* spores.
(A)** To enhance the hydrophobicity of the CW wax surface, the wax layer was heated above its meting point and *Lycopodium* spores were distributed across its surface (CW+LYCsurf) and left to cool and harden trapping the spores. This treatment initially increased the contact angle to ~130° (left, surface with *Lycopodium* spores). However, after exposure to 100 water droplets (30 µL each), the spores were washed away, leading to a noticeable decrease in contact angle to 108° (right). **(B)** In contrast, the operculum beeswax (OBW) surface without *Lycopodium* spores maintained its morphology and contact angle of 106° even after exposure to more than 11,000 water droplets, demonstrating its excellent surface stability also reflected in the stability of generated voltage peaks (see Fig. S4).


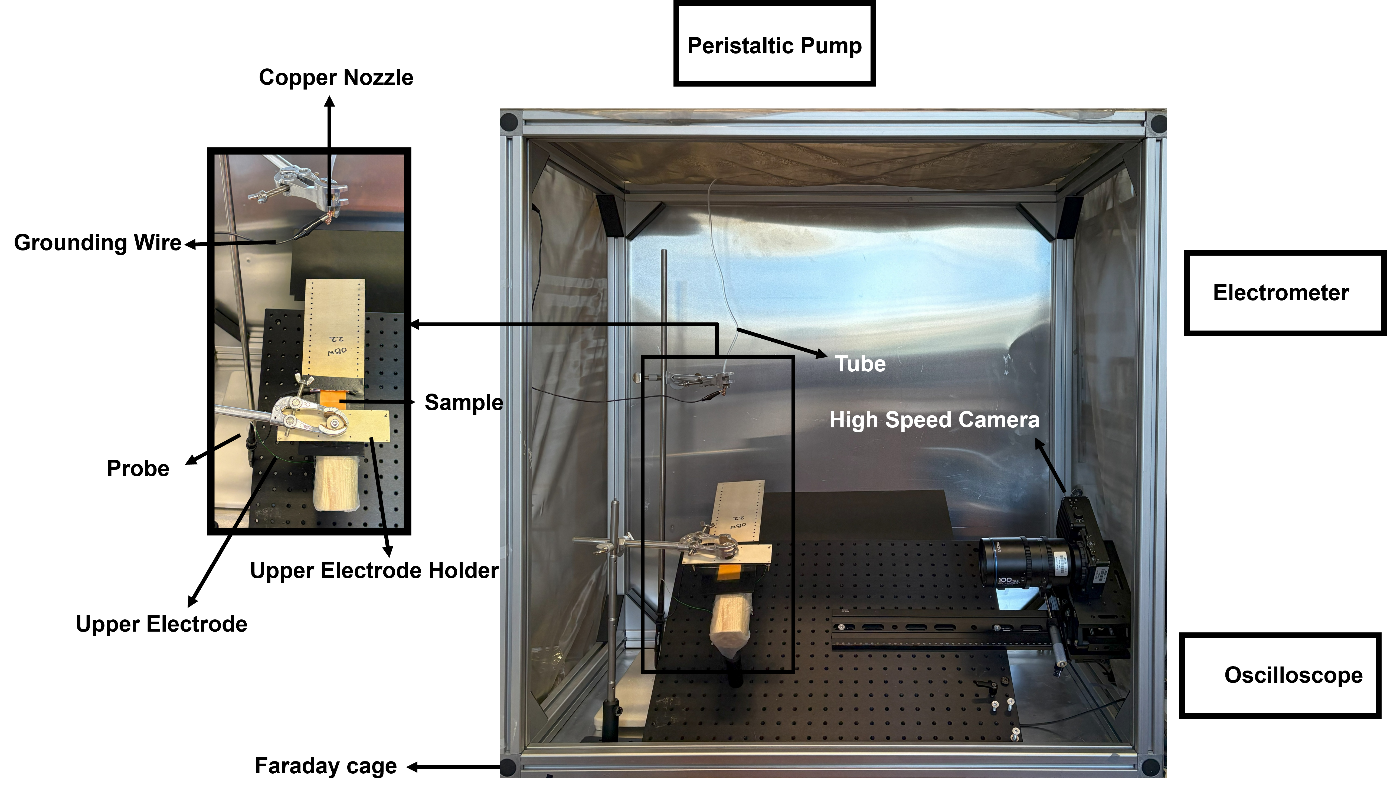

**Figure S3. Overview of the experimental setup for droplet-based triboelectric measurements.** Further details are given in the Methods section.


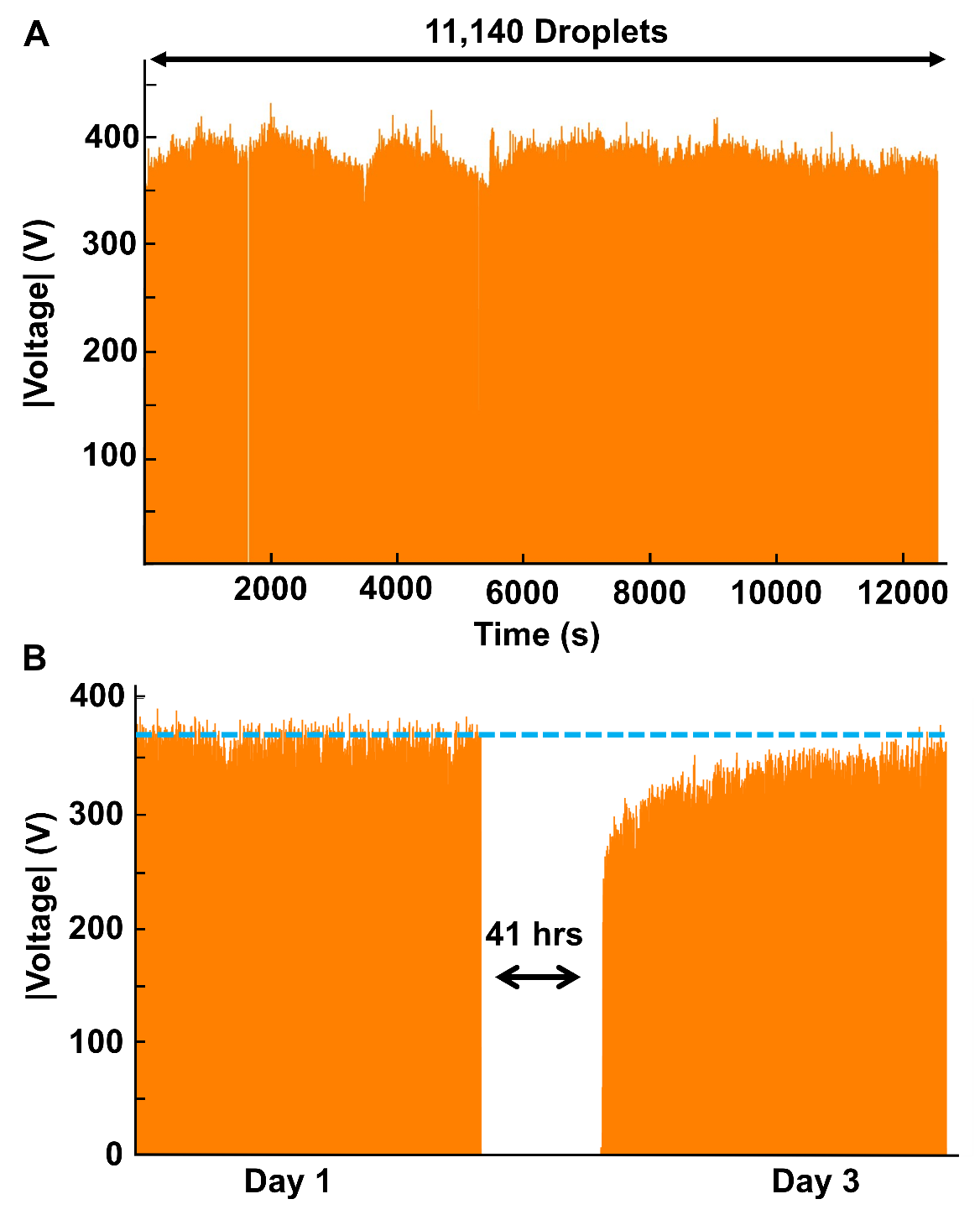


**Figure S4. Extended stability and recovery of the voltage generation on the OBW surface.** **(A)** Continuous droplet impact test showing consistent voltage output after exposure to over 11,000 water droplets (each peak corresponds to a single droplet), confirming a repeated long-term stability of the energy conversion capability of the OBW surface. **(B)** The same sample re-tested after 41 hours. After an initial recovery phase in which the voltage drop-wise increases, the voltage output finally reaches its initial level, demonstrating the excellent stability of OBW energy conversion capability. For day1 and day 3, voltage peaks of 640 droplets are shown.


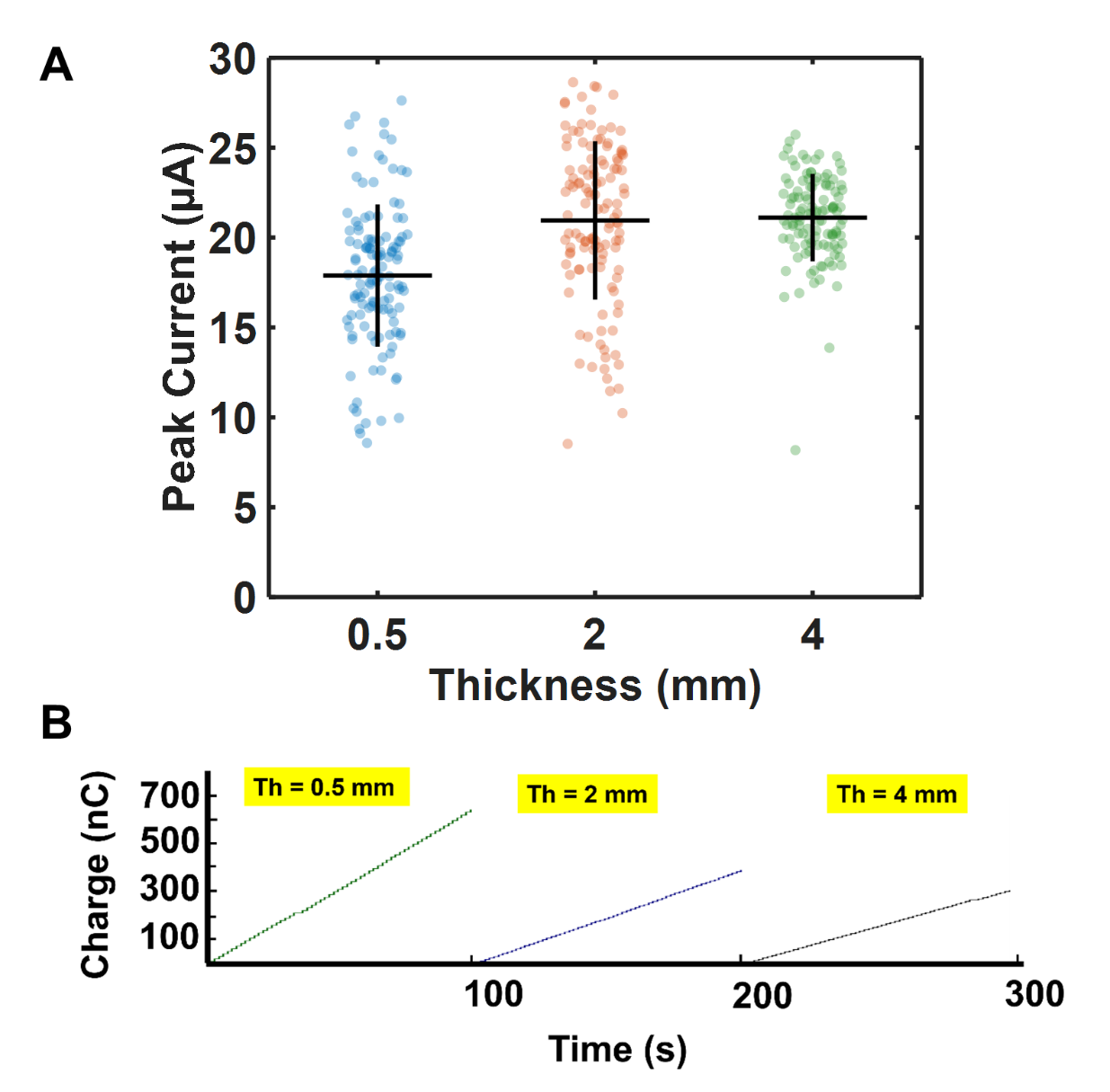


**Figure S5. Effect of OBW layer thickness. A)** Current, and B) cumulative charge signals for OBW samples with different thicknesses: 0.5, 2, and 4 mm. The test was conducted using a water pump to control the release of the water droplet (V = 40µL, n= 100 droplets) onto the samples precisely. By integrating the current peaks, the charge corresponding to each peak was calculated and reported as cumulative charge.


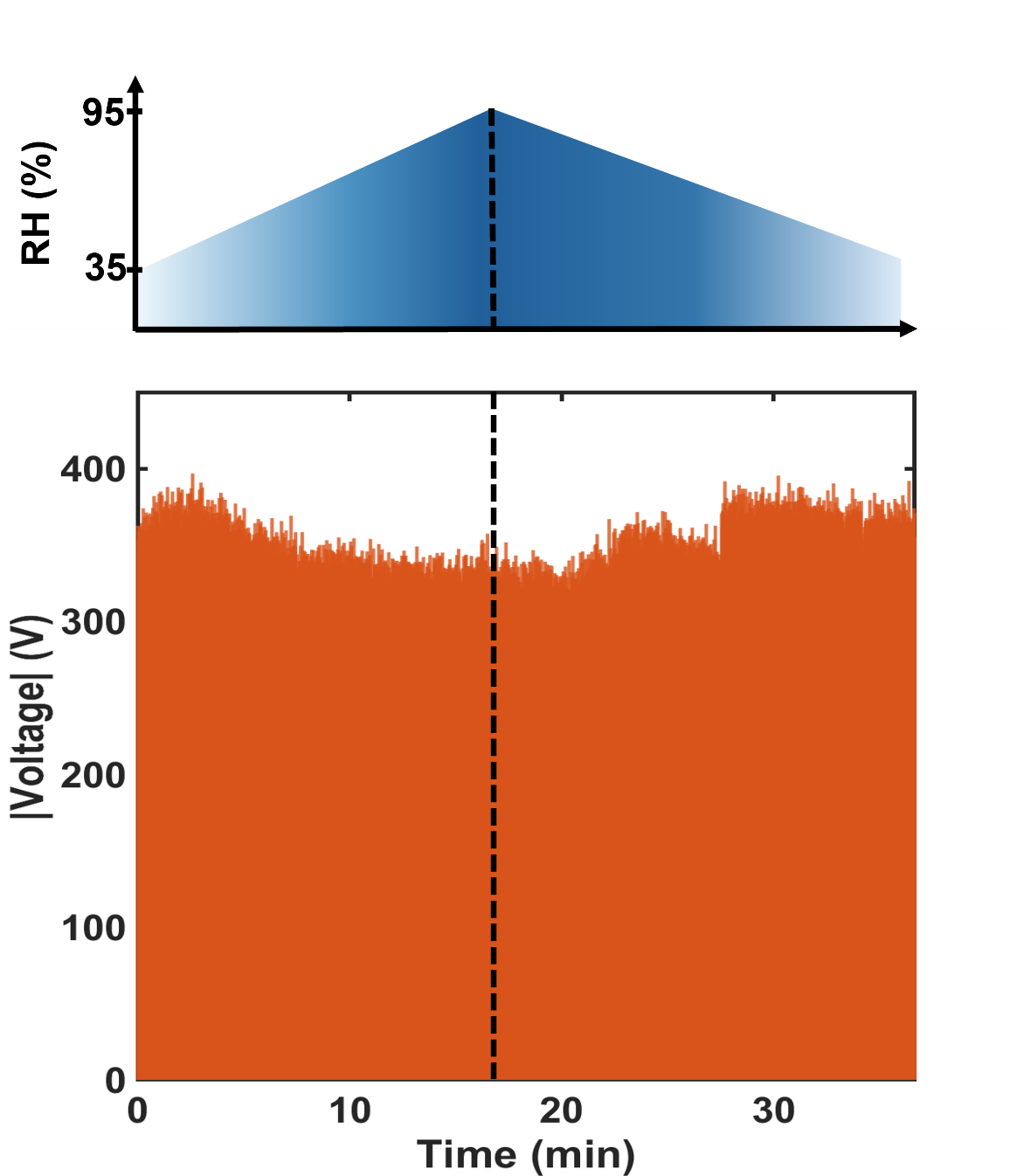


**Figure S6. Effect of continuous humidity variation on voltage output under droplet impact.** Voltage output of the OBW surface exposed to water droplets (30 µL) falling from 20 cm height during a continuous humidity sweep from 35% to 95% RH and back to 35% RH. The upper graph shows the set humidity profile. Droplet frequency was 1Hz, each peak represents a single droplet.


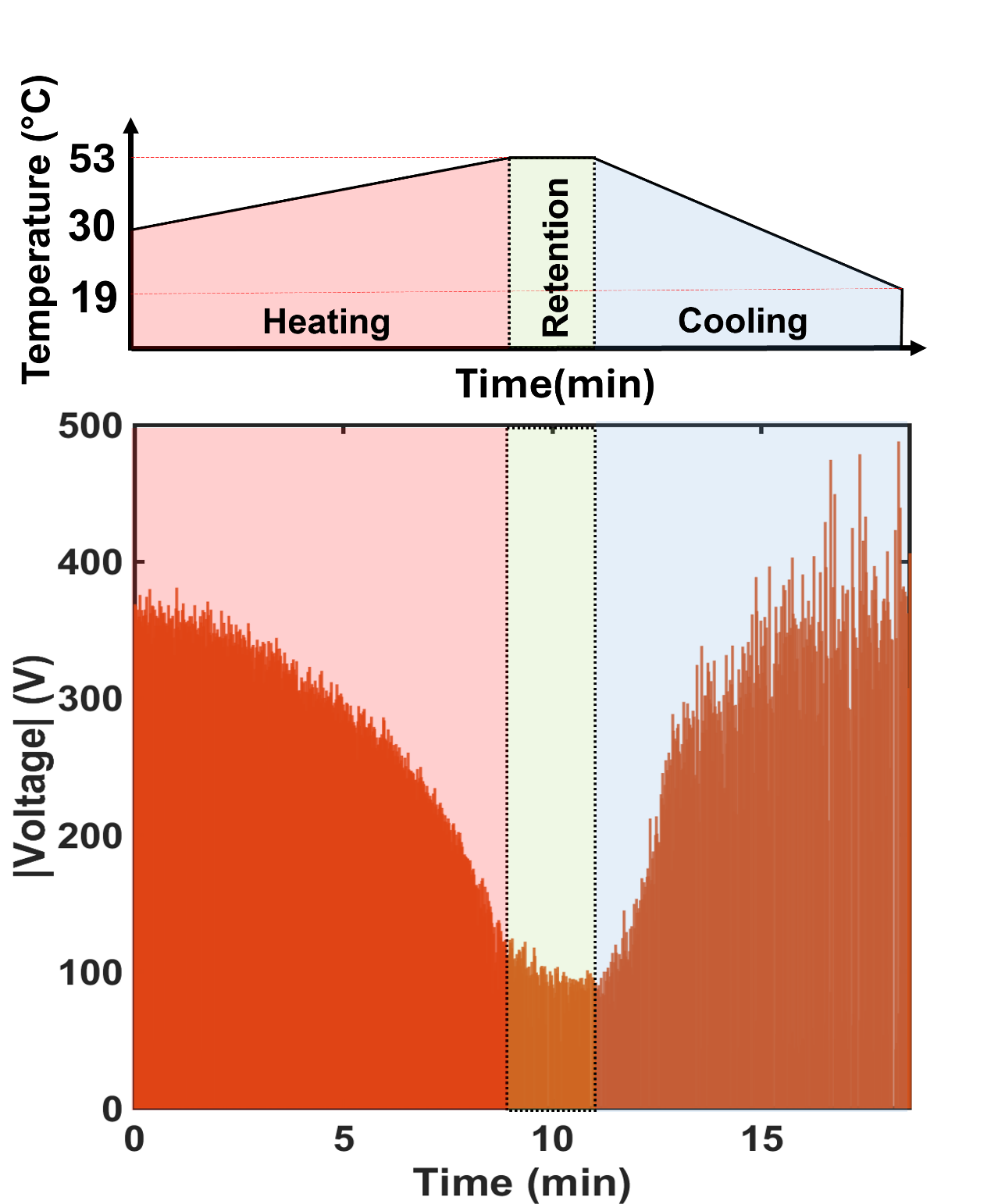


**Figure S7. Effect of temperature variation on voltage output under droplet impact.** Voltage output of the OBW surface exposed to water droplets (30 µL) falling from 20 cm height during a continuous temperature sweep from 30 °C to 53 °C ambient temperature, followed by a short retention phase and subsequent cooling to 19 °C (temperature profile given in upper panel). The water was at a temperature of ~22°C. Droplet frequency was 1Hz, each peak represents an individual droplet. The voltage amplitude decreased during heating and recovered during cooling, indicating that the charging capability of the OBW surface is affected but the effect is reversible and remains stable under transient thermal conditions.


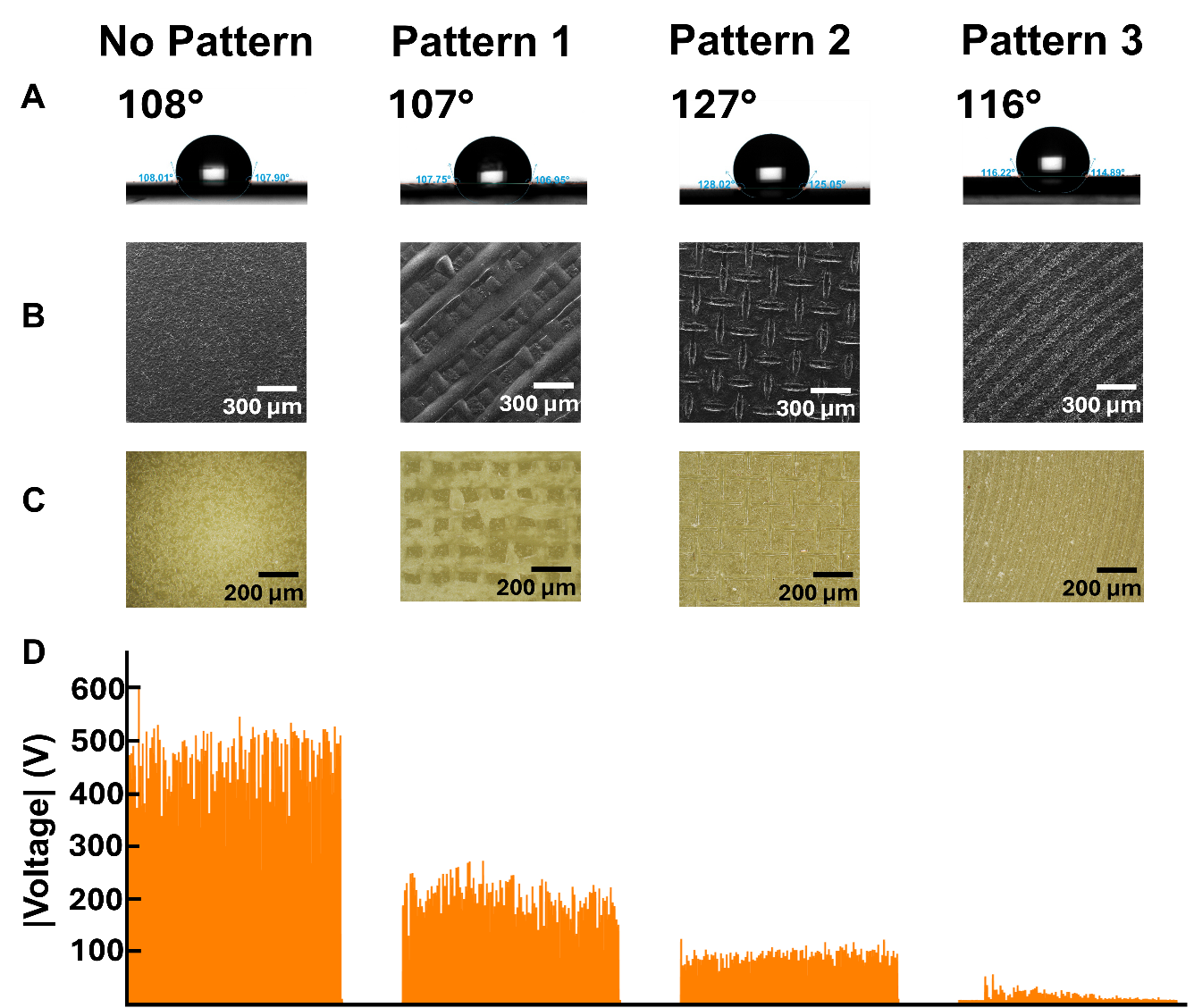


**Figure S8. Different methods were applied on the surface of the OBW samples to create microstructures.** The first method was gently melting the waxes by laser patterning vertical and horizontal lines creating a structure with rectangular pillars. The second and third method was creating different patterns by replica molding to create Patterns 2 and 3, respectively. (A) the results of the contact angle analysis for the three methods were 107°, 127°, 116°, respectively, while it was 108° for the pristine OBW surface. (B) Top-view SEM images of the different patterns. (C) Digital microscopy images of the different patterns. (D) Droplet-generated voltage peaks on pristine and patterned surfaces. A decrease in the electrical output was observed after patterning, likely due to effects occurring during complex dynamics when the droplet hits the surface, partly related to a roughness-induced droplet splashing and pinning of smaller droplets on the surface, which disrupt uniform spreading, rebounding, and droplet shedding that are essential for efficient charge transfer. Droplet frequency was 1Hz, each peak represents a single droplet.

**
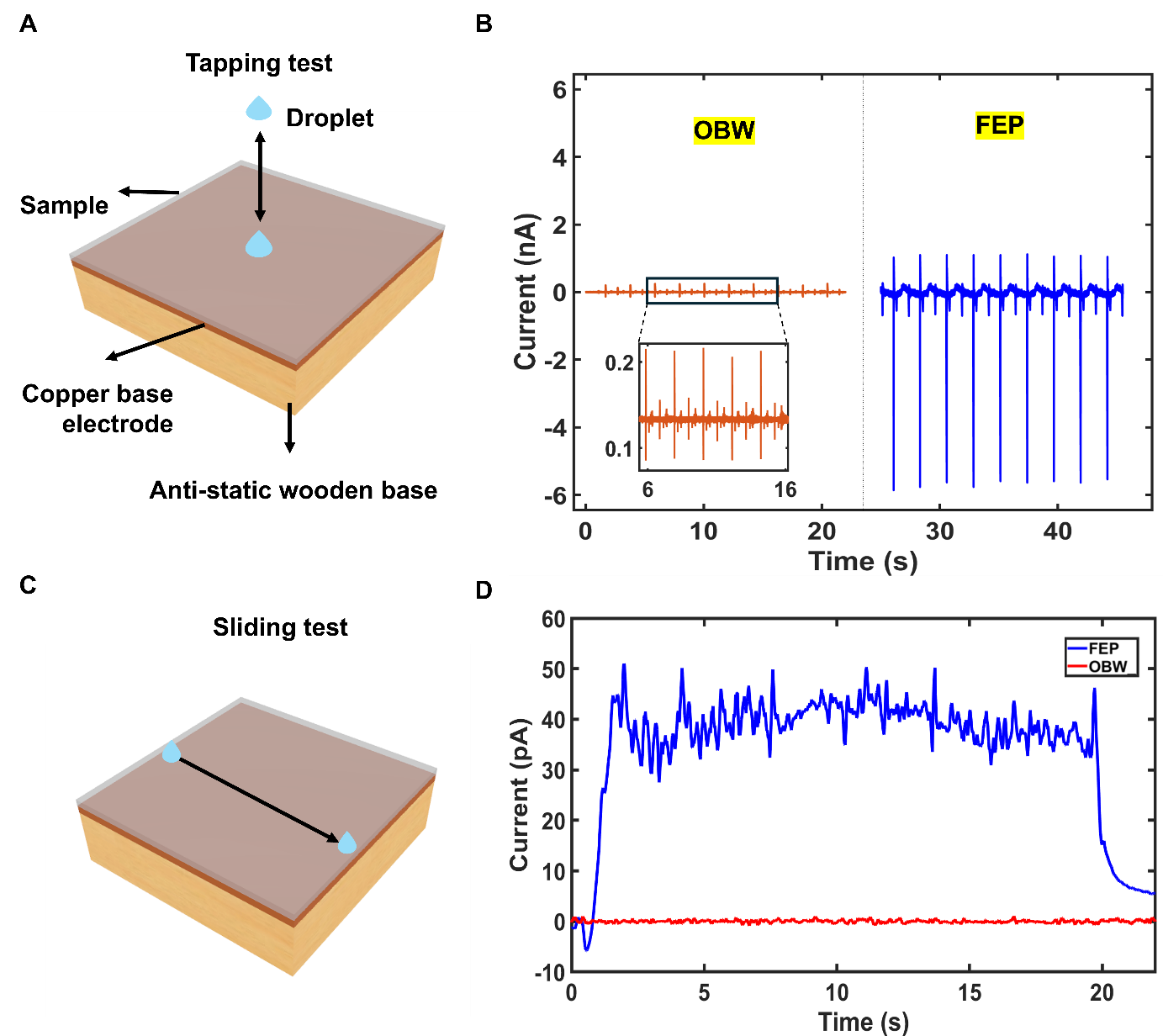
**

**Figure S9**. Contact-mode triboelectric response of OBW and FEP. (A) Schematic of the tapping test, where a neutralized water droplet is repeatedly brought into contact with and retracted from the sample surface at speed of 5mm/s while recording the transient current. (B) Representative current signals during tapping: OBW shows only small positive peaks (~200 pA), whereas FEP exhibits pronounced negative spikes (up to –6 nA) at each droplet impact, confirming a strong triboelectric response under small contact forces. (C) Schematic of the sliding test, where a droplet is translated ~20 mm across the surface at 1 mm/s to generate continuous contact. (D) Current response during sliding: FEP (blue) produces a stable current plateau (~30–50 pA) during droplet motion, returning immediately to baseline after sliding stops. OBW remains at baseline throughout, indicating no measurable response. These results demonstrate that in contrast to FEP, which responds under both gentle tapping and sliding contact forces, OBW requires high-energy droplet impacts to generate charges.


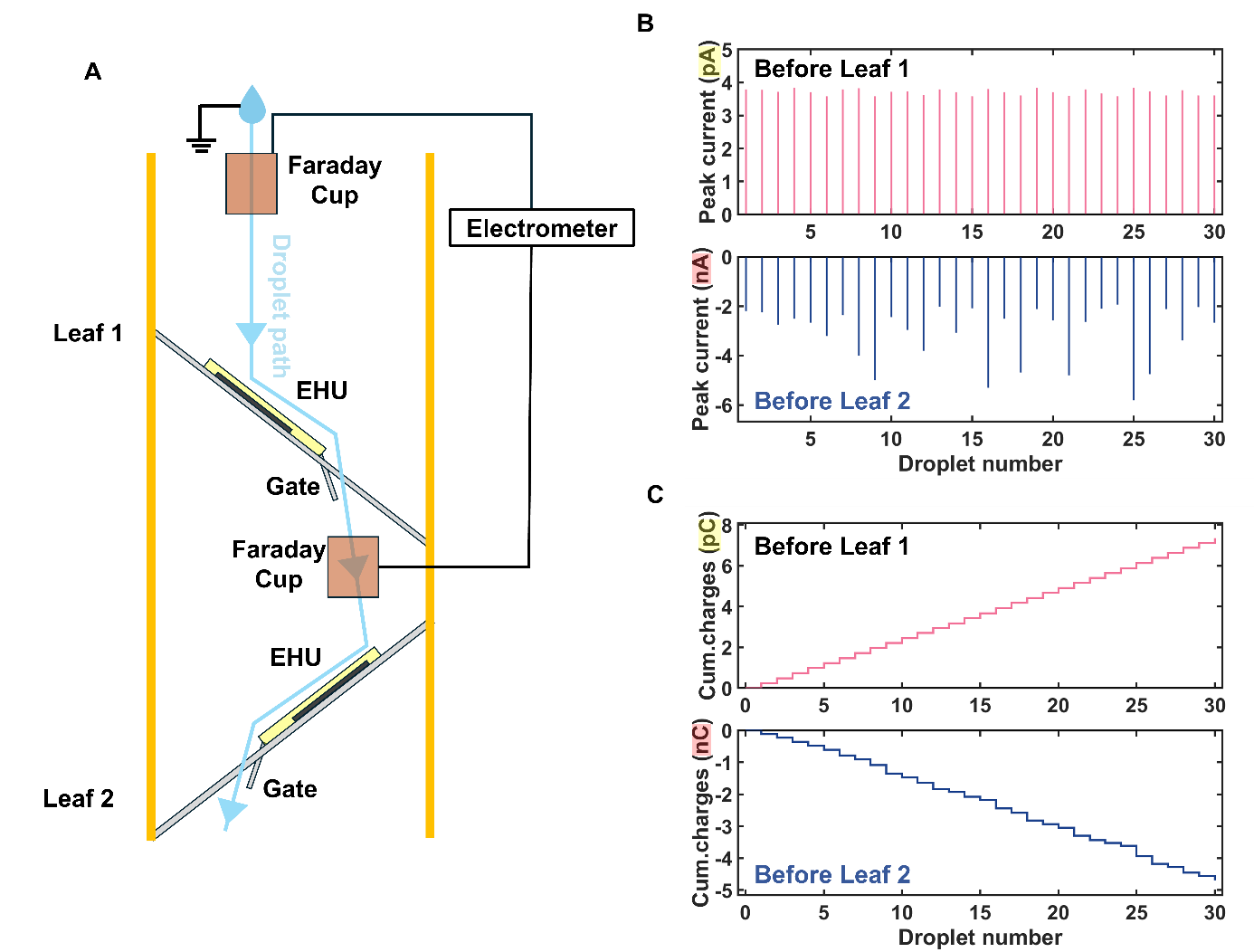


**Figure S10. Charge state of droplets before Leaf 1 and before Leaf 2.** (A) Experimental setup for charge measurements. Droplets were grounded upon leaving the nozzle and then collected in custom Faraday cups connected to an electrometer, positioned either before Leaf 1 or before Leaf 2. (B) Peak current signals of individual droplets measured at the two positions. Before Leaf 1, for 30 droplets exhibit very small positive currents in the pA range (yellow), consistent with nearly neutralized droplets. Before Leaf 2, droplets show significantly larger negative peaks in the nA range (pink), confirming that they are charged after interacting with Leaf 1. (C) Corresponding cumulative charge of multiple droplets, each step represents an individual droplet passing the Faraday cup. Before Leaf 1, the cumulative charge remains in the low pC range, whereas before Leaf 2, cumulative charge reaches significantly higher values in the nC range.


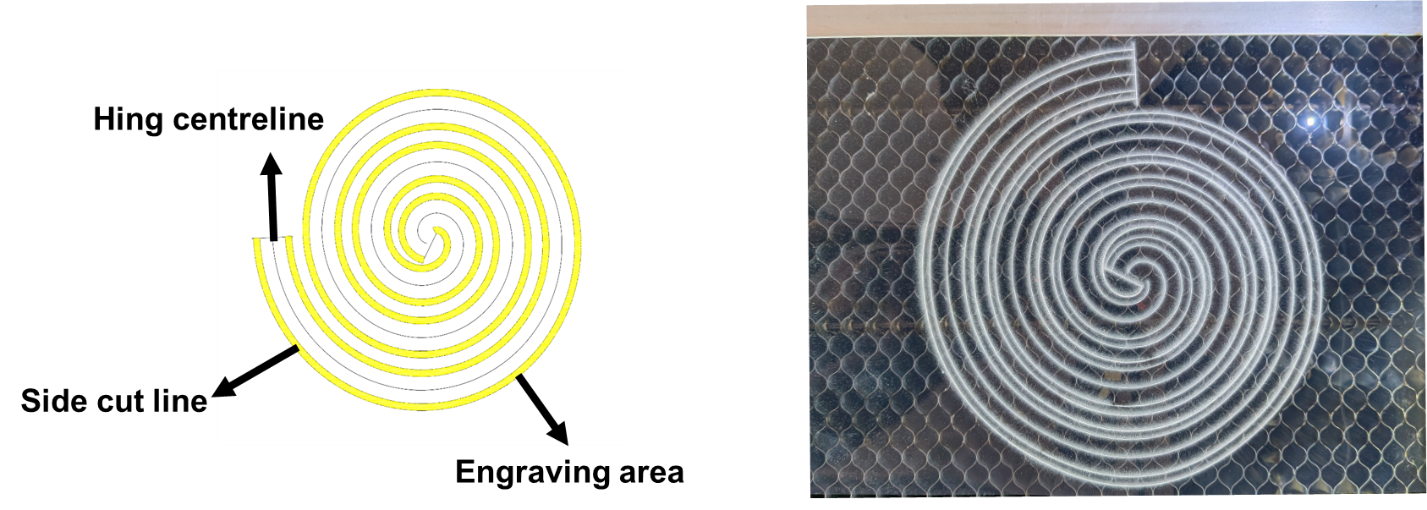


**Figure S11. Single‐step laser‐cut fabrication of spiral prototypes** Left) CAD layout: Continuous centreline (black): laser-engraved crease for folding the FEP sheet into a V-shaped profile. Hatched regions (yellow): areas of fine engraving to produce sub-surface microstructures, boosting surface hydrophobicity. Perimeter cutline (gray): through-cut path to release the spiral element from the sheet. Right (Photograph of laser-cut part): The same spiral geometry, engraved and cut in a single pass on a cellulose acetate substrate.

**Table S1. Market prices of FEP and OBW in Europe and North America (as of September 2025), listed by thickness/shape and supplier.**

| FEP | OBW | Supplier |
| --- | --- | --- |
| 6270 €/kg (thickness = 0.1 mm) 2448 €kg (thickness = 0.5 mm) 1244 €/kg (thickness = 1 mm) | 254-372 **€**/kg | Sigma-Aldrich[^1^](https://www.sigmaaldrich.com/IT/it/product/aldrich/243248)^,^[^2^](https://www.sigmaaldrich.com/IT/it/product/aldrich/243221)^,^[^3^](https://www.sigmaaldrich.com/IT/it/product/aldrich/gf11411086)^,^[^4^](https://www.sigmaaldrich.com/IT/it/product/aldrich/gf13265388)^,^[^5^](https://www.sigmaaldrich.com/IT/it/product/aldrich/gf69874037) |
| 640 €/kg (0.8 mm – tube shape) | 70 **€**/kg | Carl Roth[^1^](https://www.carlroth.com/com/en/a-to-z/beeswax-white/p/5825.2)^,^[^2^](https://www.carlroth.com/com/en/hoses-for-general-applications/tube-rotilabo-fep/p/c022.1) |
| 2957 $/kg (0.001 in = 0.0025 mm) | 107 $/kg | Fisher Scientific[^1^](https://www.fishersci.com/shop/products/beeswax-9/S25192A)^,^[^2^](https://www.fishersci.com/shop/products/1mil-fep-film-typea-24in-by-ft/NC3588781#?keyword=FEP%20film) |

1. **Supplementary Videos**

**Movie 1.** The video illustrates the adhesion of operculum beeswax (OBW) coatings on various substrates, i.e. copper, zinc, and cellulose acetate. The OBW layer forms a uniform and stable coating on all tested surfaces, maintaining good interfacial contact and mechanical integrity during deformation.

**Movie 2.** The video compares the flexibility of various coated materials: FEP, OBW, CBW, CW, CW+LYC, and PINE applied on copper tape. FEP, OBW, and CBW exhibit high flexibility and remain well-adhered to the substrate during bending. In contrast, CW and CW+LYC films crack and partially detach under deformation, while PINE becomes rigid and brittle and fractures under applied force.

**Movie 3.** Droplet impact and spreading on the samples surface was recorded at 2408 fps videos using a high-speed camera to detect the maximum spreading diameter of the droplet. The corresponding frame in which the droplet reaches to the maximum length on the surface, is exported from the software and used for measurements and comparison. For each material, 9 measurements have been performed. This video corresponds to FEP, OBW, CW, CW+LYC, and Pine, respectively.

**Movie 4.** High-speed videos comparing the maximum spreading diameter of water droplets with different volumes impacting the OBW surface. The recordings reveal the increase in spreading diameter with droplet volume, consistent with the quantitative data presented in Figure 3G.

**Movie 5.** This video shows a real-time recording of water droplets splashing continuously on the surface of the OBW sample, with 100 LEDs lighting up instantly upon each droplet impact.

**Movie 6.** This video shows a real-time recording of water droplet interaction with the preliminary prototype composed of two cellulose acetate artificial leaves with zinc as the bottom electrode, OBW as the triboelectric material, and a wire as the top electrode. To control the test, a water droplet is released onto the first leaf every second using a peristaltic pump. When the droplet splashes on the surface of OBW and contacts the top electrode (on the first leaf), the LEDs light up. Guided by the droplet structure, it subsequently passes through the gate hole to the next leaf. A similar process occurs on the second leaf, where contact with the second top electrode results in a second LED activation.

**Movie 7.** High-speed videos show the droplet impact locations on Leaf 1 and Leaf 2 of the prototype. On Leaf 1, droplets consistently land in nearly the same area, whereas on Leaf 2 the landing positions vary significantly. This difference arises from the less precise droplet-guiding mechanism of Leaf 2’s gate design, which could be addressed in future prototypes.

**Movies 8 and 9.** To advance the prototype model, we developed a spiral model that can be hung from the end of the plant leaves. These videos show real-time recordings of water droplet interaction with two different spiral designs made with FEP and cellulose acetate. The video with FEP shows a spiral model with engraved microstructrued edges (superhydrophobic to guide the droplet and avoiding bouncing off the spiral). In cellulose acetate model, two holes were designed to guide the droplet down to the next coil of the spiral and take advantage of its impact for energy harvesting.
